# The Kinetics of Carbon‐Carbon Bond Formation in Metazoan Fatty Acid Synthase and Its Impact on Product Fidelity

**DOI:** 10.1002/anie.202412195

**Published:** 2024-12-04

**Authors:** Christian Gusenda, Ana R. Calixto, Joana R. Da Silva, Pedro A. Fernandes, Martin Grininger

**Affiliations:** ^1^ Institute of Organic Chemistry and Chemical Biology Buchmann Institute of Molecular Life Sciences Goethe University Frankfurt Max-von-Laue-Str. 15 60438 Frankfurt am Main Germany; ^2^ LAQV, REQUIMTE Departamento de Química e Bioquímica Faculdade de Ciências Universidade do Porto Rua do Campo Alegre s/n 4169-007 Porto Portugal

**Keywords:** biocatalysis, fatty acids, biosynthesis, protein-protein interactions, molecular dynamics

## Abstract

Fatty acid synthase (FAS) multienzymes are responsible for de novo fatty acid biosynthesis and crucial in primary metabolism. Despite extensive research, the molecular details of the FAS catalytic mechanisms are still poorly understood. For example, the β‐ketoacyl synthase (KS) catalyzes the fatty acid elongating carbon‐carbon‐bond formation, which is the key catalytic step in biosynthesis, but factors that determine the speed and accuracy of his reaction are still unclear. Here, we report enzyme kinetics of the KS‐mediated carbon‐carbon bond formation, enabled by a continuous fluorometric activity assay. We observe that the KS is likely rate‐limiting to the fatty acid biosynthesis, its kinetics are adapted to the length of the bound fatty acyl chain, and that the KS is also responsible for the fidelity of biosynthesis by preventing intermediates from undergoing KS‐mediated elongation. To provide mechanistic insight into KS selectivity, we performed computational molecular dynamics (MD) simulations. We identify positive cooperativity of the KS dimer, which we suggest to affect the conformational variability of the multienzyme. Advancing our knowledge about the KS molecular mechanism will pave the ground for engineering FAS for biotechnology applications and the design of new therapeutics targeting the fatty acid metabolism.

## Graphical abstract

The β‐ketoacyl synthase (KS) domain of metazoan fatty acid synthase catalyzes the condensation reaction of an acyl and malonyl substrate with two cooperatively working subunits. The KS ensures the fidelity of the fatty acid biosynthesis i) by increasing the speed with substrate length and ii) by discriminating against the intermediates of the fatty acid cycle.

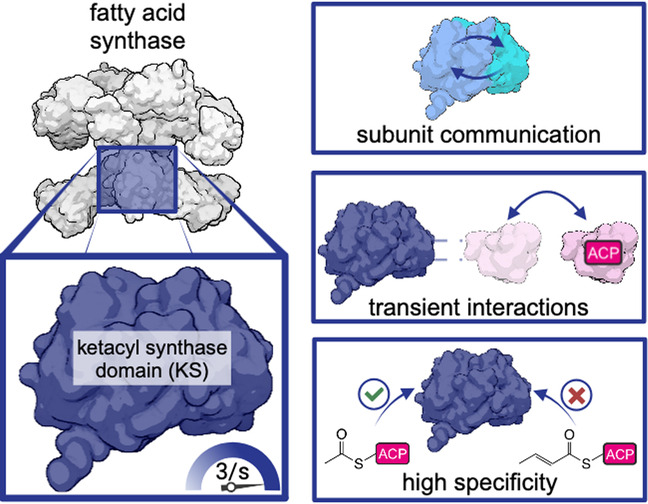

## Introduction

Fatty acids are essential for life. They are signaling molecules, components of membrane‐building phospholipids, esterified to triacylglycerols for energy storage, and stabilize or localize other molecules for cellular functions.[^1^, ^2^] Fatty acids are de novo biosynthesized by fatty acid synthases (FASs) by condensation and processing of the activated carbonic acids, acetyl‐ and malonyl‐coenzyme A (CoA), to long‐chain fatty acids, most commonly palmitic acid (C16) or stearic acid (C18).^[3]^ Advancing the knowledge of FASs is important due to the fundamental role they play in primary metabolism. In addition, FAS presents a promising target for treating a wide range of diseases, like obesity and cancer,[^4^, ^5^, ^6^, ^7^, ^8^, ^9^] and a better mechanistic understanding of the FAS molecular properties can lay the foundation for new therapeutic strategies.

**Figure 1:**
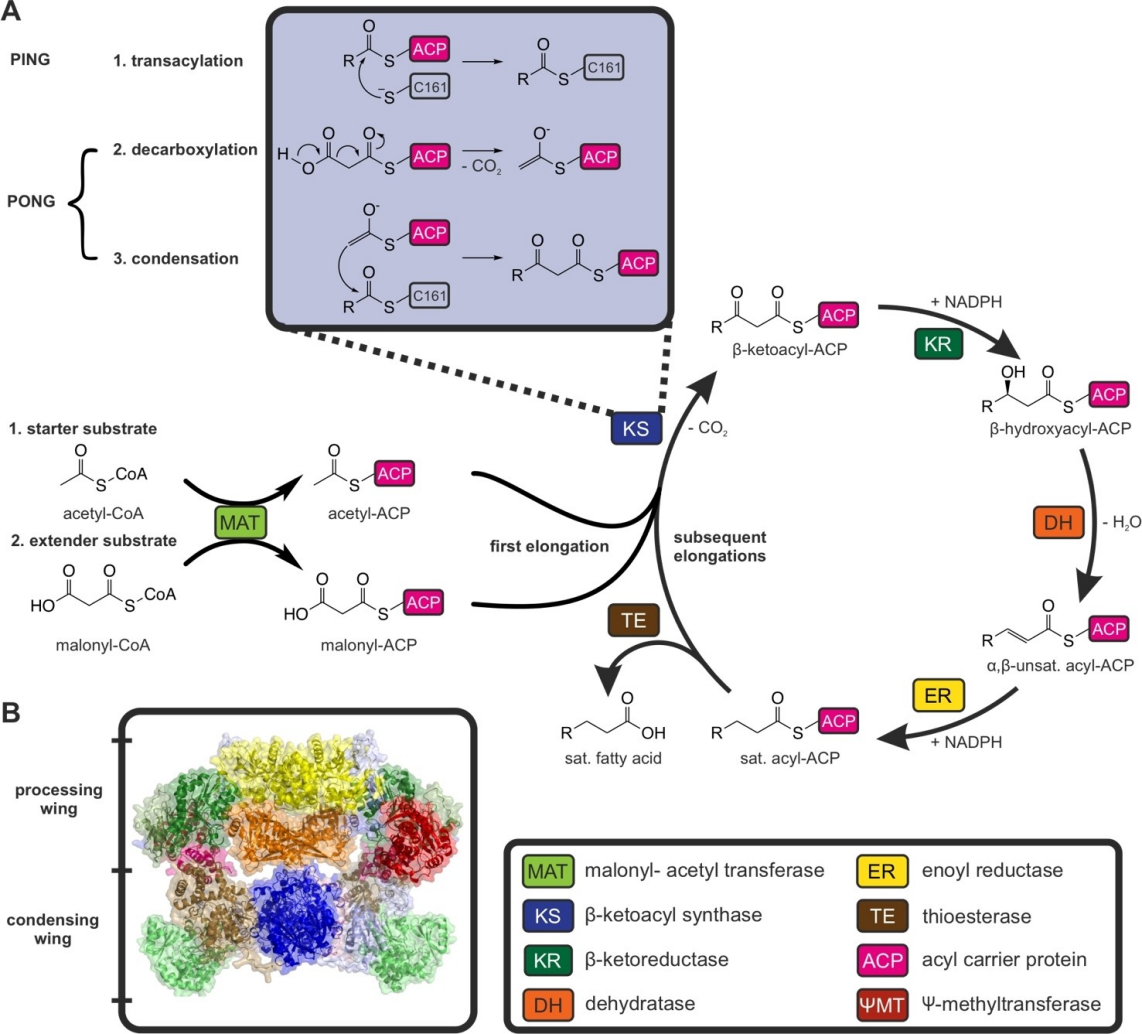
Mechanism of the fatty acid synthesis. **A** The acetyl moiety of acetyl‐CoA is transferred to an acyl carrier protein (ACP) by a malonyl‐acetyl transferase (MAT) catalyzed reaction, and subsequently loaded onto the active side cysteine of the β‐ketoacyl synthase (KS) (1. Transacylation). Free ACP is equipped with a malonyl moiety derived from the extender substrate malonyl‐CoA. Malonate is decarboxylated to an enolate (2. Decarboxylation), which acts a nucleophile in a Claisen condensation reaction with the KS bound acetyl (3. Condensation). The β‐ketoacyl condensation product, that is bound to ACP, is transferred to the β‐ketoreductase (KR), which reduces the keto group to a hydroxy group, to the dehydratase (DH) for dehydration, and to the enoyl reductase (ER), which reduces the unsaturated acyl‐ACP to the saturated compound. The cycle is repeated until a chain length of typically 16 carbon atoms is reached (palmitic acid). The thioesterase (TE) catalyzes the hydrolysis of the product from the ACP, which releases the free fatty acid. We note that, following work of Witkowski et al., bicarbonate instead of CO_2_ is released during decarboxylation reaction.^[38]^ **B** Structure of murine fatty acid synthase from the alpha fold database (AFDB: AF−P19096‐F1) with indication of condensing wing (KS, MAT) and processing wing (KR, DH, ER, ΨMT). The processing wing includes a catalytically inactive KR subunit (ΨKR) and a catalytically inactive methyltransferase (ΨMT).

While sharing the same biosynthetic principle (Figure 1A), FASs are divided by their overall structural organization into two main systems: type I and type II FASs. Type II FASs are mostly present in bacteria, plants and eukaryotic organelles of prokaryotic descent. This type consists of single, monofunctional proteins, which are encoded by discrete genes.^[10]^ In contrast to this, type I FASs are multi‐enzyme proteins in which catalytic domains are covalently linked to each other (Figure 1B). The overall architecture of type I FAS in metazoa is identical in the related polyketide synthases (PKS), which additionally share the same reaction cascade but produce a higher variety of products.[^11^, ^12^, ^13^, ^14^, ^15^, ^16^, ^17^, ^18^] The concept of multi‐enzyme complexes brings advantages such as high effective concentrations, which lead to high turnover rates and reduction of side reactions by direct substrate shuttling. Moreover, fusing several genes into a single gene encoding a multidomain protein reduces the need for complex, separate regulatory networks, enabling fast and synchronized responses to environmental changes. The evolutionary fusion of discrete type II enzymes to domains in type I systems probably induced kinetic alterations that are not yet fully understood.

The structure of the metazoan FAS was determined in 2006 with the X‐ray structure of pig FAS that revealed an x‐shaped domain architecture. Since then, structural insight has been further refined with additional X‐ray, cryogenic electron microscopic data and molecular modelling.[^3^, ^19^, ^20^, ^21^, ^22^, ^23^] These findings, coupled with detailed biochemical studies, offer a detailed understanding of the iterative synthesis by metazoan FASs. Remarkably, metazoan FASs operate at extremely high rates, up to two orders of magnitude faster than related PKSs. The turnovers of the single domains of the enzyme complex were studied previously.[^24^, ^25^, ^26^, ^27^, ^28^, ^29^, ^30^, ^31^, ^32^, ^33^, ^34^, ^35^, ^36^] These studies identified the KS catalyzed decarboxylation reaction as one of the slowest reactions but were unable to identify a unique rate‐limiting step of the fatty acid biosynthesis.^[4]^

**Figure 2:**
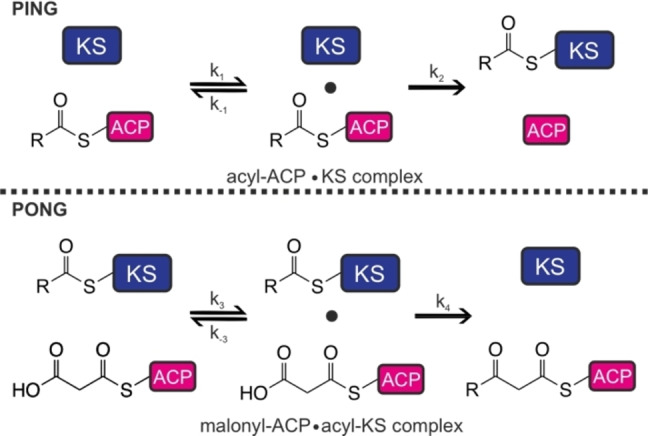
Overall reaction of KS‐mediated chain elongation split into transacylation (ping step) and decarboxylative condensation (pong). During the ping step, an enzyme‐substrate complex is formed and exists in equilibrium with the dissociated enzyme and substrate. The rate with which the complex forms is called k_1_. The rate k_−1_ describes the complex dissociation rate. The transacylation of the acyl moiety to the KS Cys161 and the subsequent dissociation of the enzyme‐product complex is governed by the rate constant k_2_. During the pong step, the enzyme‐substrate complex of acyl KS and malonyl‐ACP is formed with the associating rate of k_3_ and the dissociating rate of k_−3_. The decarboxylation, condensation reaction and dissociation of the enzyme‐product complex are subsumed by the rate constant k_4_.

**Figure 3:**
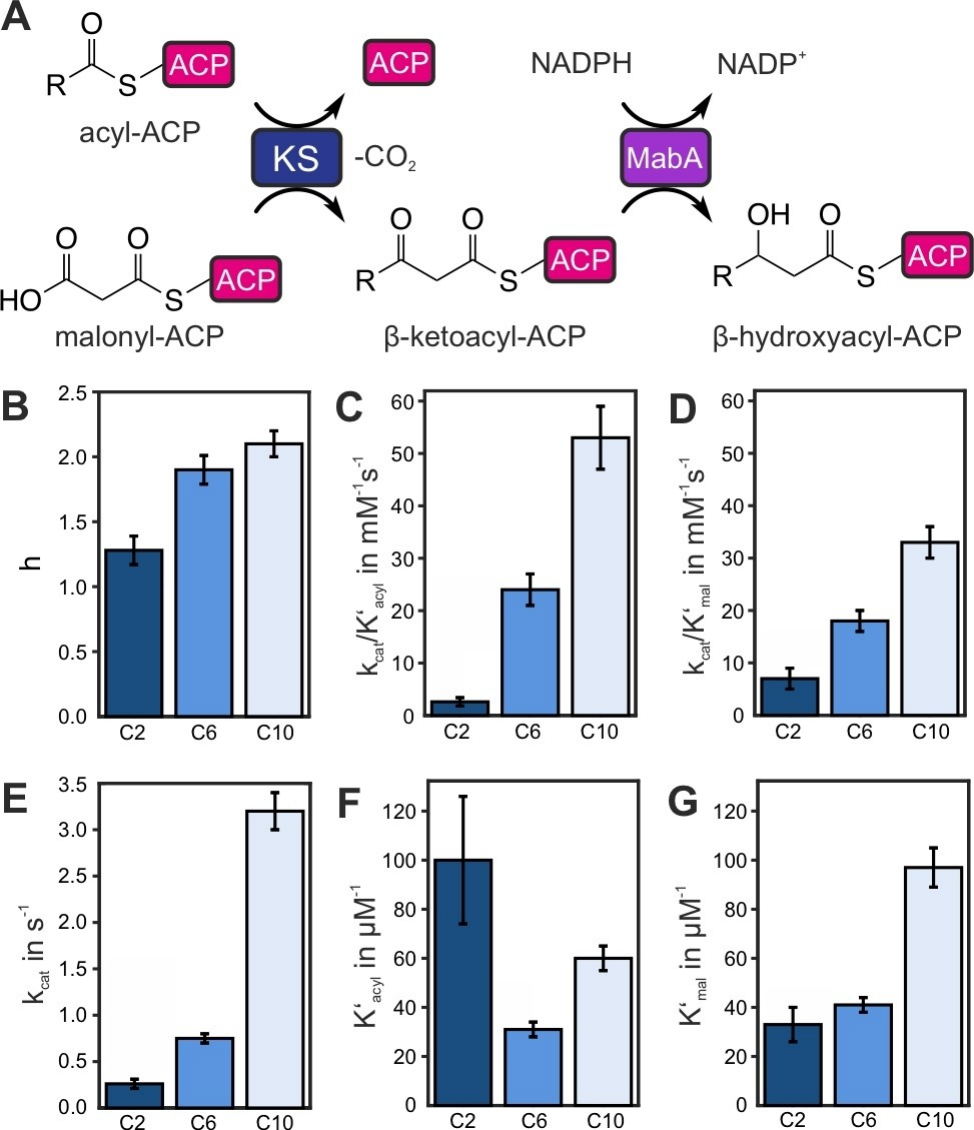
Kinetic analysis of the wildtype KS. **A** Scheme of the MabA‐coupled assay. The MabA‐coupled assay allows continuous monitoring of the entire KS‐mediated two‐step condensation reaction by the NADPH‐dependent reduction of the condensation product. Maintaining an excess reduction capacity with high concentrations of reductase allows to indirectly monitor the formation of β‐keto acyl‐ACP and, therefore, the KS activity. The formation of the β‐keto acyl product is proportional to the oxidation of the reducing agent NADPH and, therefore, to NADPH fluorescence quenching. **B** Hill constant h of the wildtype KS for acetyl‐ACP, hexanoyl‐ACP and decanoyl‐ACP; **C** Enzymatic efficiency *k*_cat_/K′_acyl_ regarding the ping step; **D** Enzymatic efficiency *k*_cat_/K′_mal_ regarding the pong step; **E** Turnover number *k*_cat_; **F** Michaelis–Menten constant derived value K′_acyl_ regarding acyl‐ACP; **G** Michaelis–Menten constant derived value K′_mal_ regarding malonyl‐ACP. Bar heights represent the means of three biological replicates, error bars show standard deviations. All measurements were performed at 25 °C.

Aiming to understand the remarkable metazoan FAS catalytic efficiency, the KS domain deserves a closer look, particularly, as it catalyzes the pivotal carbon‐carbon bond formation during fatty acid synthesis. Catalysis by the KS occurs via a sequential ping‐pong mechanism (Figure 2).^[4]^ The first step is the transacylation part of the reaction, which involves the transfer of an acyl from ACP to the active site residue Cys161 (murine FAS numbering is used throughout this article). The second half‐reaction is the decarboxylation of the elongating malonyl residue and its Claisen condensation with the Cys161‐bound acyl residue (Figure 1A). So far, only the kinetics of the transacylation and the decarboxylation part reactions of metazoan FAS have been studied.[^25^, ^37^, ^38^] However, in probing specific portions of KS catalysis, studies were unable to capture features of the overall reaction; e.g., if and how the KS kinetics is adapted to the chain length of the substrate. In addition, these studies failed to reveal key details, including for example the KS/ACP interactions that have been suggested to be a key factor in orchestrating biosynthesis in type I and II fatty acid and polyketide biosynthesis.[^39^, ^40^, ^41^, ^42^, ^43^, ^44^, ^45^] The mechanistic details of the condensation reaction were identified previously.^[38]^ However, the kinetics of the condensation reaction is poorly characterized and has so far focused on bacterial type II KS. ^14^C‐labelled malonyl‐CoA was used in the first study of bacterial KS.^[46]^ Later, an enzyme‐coupled assay enabled continuous monitoring of the full KS‐catalyzed reaction. In this case, stand‐alone β‐ketoacyl reductases are harnessed to detect product formation by spectral changes upon consumption of the cofactor NADPH (Figure 3A).[^47^, ^48^] Several more functional and structural studies were conducted on bacterial KS enzymes.[^49^, ^50^, ^51^, ^52^, ^53^]

In the present study, we asked whether the KS‐mediated condensation reaction affects the kinetics and specificity of the overall elongation reaction. For this, we determined the full‐reaction kinetics of the type I mFAS (metazoan FAS)‐KS domain for ACP‐bound substrates. To do so, we employed an enzyme‐coupled assay (Figure S1) to characterize the kinetics of the KS domain for three natural substrates, which revealed chain length specificity of the enzyme, as well as for non‐natural substrates, which indicated that the KS is responsible for the fidelity of fatty acid biosynthesis. The experimental insight into substrate discrimination was complemented by molecular modelling. Compared to saturated fatty acids, the in silico substrate molecular dynamics (MD) simulations revealed that the fatty acid cycle intermediates bind to the KS binding tunnel in catalytically unproductive positions. With the help of the enzyme‐coupled assay, we further identified the cooperative working mode of the KS dimer. Although the molecular basis for the positive cooperativity remains elusive, we demonstrate that domain‐domain interactions and the particular combination of starting and elongating substrates are relevant to the cooperative response.

## Results and Discussion

### Enzyme Coupled Fluorometric Assay

We began this study with the question of whether the responsibility for maintaining high reaction rates, while ensuring accuracy in terms of product chain length and chemistry, might lie with the KS domain through its selection of appropriate substrates for condensation. To understand how the KS domain could accomplish this complex task, we initiated the analysis of the KS enzyme's kinetic properties. We decided to work with the KS domain of the type I murine FAS because we had previously established recombinant access to the protein. The sequence of murine FAS is overall 82 % identical to human FAS, with 89 % sequence identity for the KS domain. Since the KS domain is not accessible as a stand‐alone protein in sufficient yield and quality via recombinant protein production, the KS‐MAT didomain, carrying mutation S581A (functional MAT knockout, MAT^0^) and termed KS‐MAT^S581A^ hereafter, was used in this assay (Figure S2–S3).^[32]^

The KS‐mediated condensation reaction does not involve substrates or products that can easily be monitored continuously by spectroscopic means, nor does it include consumption or production of cofactors with spectroscopically accessible properties. Therefore, when attempting to develop a direct and continuous assay as a platform for the thorough analysis of the KS kinetics, we decided to adapt the *Mycobacterium tuberculosis*‐based coupled assay for type I FAS (Figure 3A).^[47]^ The assay is based on the in vitro catalysis of the KS‐mediated condensation reaction and adds a subsequent reduction step from fatty acid biosynthesis by including the NADPH‐dependent β‐ketoacyl reductase MabA, and is therefore referred to as MabA‐assay. The reduction and hence the prior condensation can be monitored by detecting fluorescence quenching of NADPH.

The use of acyl‐ACP substrates beyond the chain length of ten carbon atoms was limited due to their decreased half‐lives (Figure S4). We sought to switch to (S, N)‐acetylcysteamine (SNAC)‐derived model substrates instead of ACP tethered substrates to circumvent acyl‐ACP stability problems (Figure S5). However, due to poor solubility at acyl chain lengths larger than six carbon atoms, these substrates proved to be unsuitable for our needs. Coenzyme A tethered substrates, on the other hand, induce substrate inhibition at low concentrations such that they are unsuitable for achieving an understanding of the KS that closely mirrors natural conditions (Figure S6–S7). Thus overall, we conducted this study with acyl‐ACPs up to an acyl chain length of C10 (Figure S8–S9).

### Determination of Kinetic Constants

The enzyme specific kinetic constants *K*_m_ and *k*_cat_ are commonly used to compare different enzymes or different substrates in one enzyme catalyzed reaction. To determine the kinetic constants of the overall two‐step condensation reaction, a starter acyl substrate and an elongation malonyl substrate were applied in different concentrations to measure the initial reaction velocity using the MabA assay (Figure S10–S11). The obtained data (initial reaction velocity against acyl‐ACP concentration) exhibited a sigmoidal shape, which indicates a cooperative behavior of the KS. To account for the deviation from a simple hyperbolic saturation curve, the data was globally fitted including a Hill coefficient to account for sigmoidal kinetics (equation 1, S2; Figure S12; Table S1). The value v_max_ reflects the maximal velocity of the enzyme. K′ is related to the Michalis‐Menten constant *K*_m_, which reflects the substrate concentration at 0.5×v_max_, but additionally K′ takes substrate occupancy and affinity into account. The Hill constant h is a measure of cooperativity in enzyme catalysis, where binding of substrate S to the first active site enhances (or decreases) the affinity of the same substrate S to other active sites. The sigmoidal response was previously observed for the transacylation half reaction of mFAS‐KS as well as for type II homologs.[^37^, ^54^] Further, we noticed that the steepness of the sigmoidal titration curve depends on the chain length of the substrates indicating that cooperativity is a function of acyl chain length (Figure 3B).

$$v=\frac{v_{max}{\left[S\right]}^{h}}{{K^{'}+\left[S\right]}^{h}}$$

The enzymatic efficiency *k*_cat_/K′ can be calculated for both the ping and the pong step of a double displacement reaction (Figure 2, equation 2.^[55]^ The catalytic efficiency of the ping step is, therefore, reflected by *k*_cat_/K′_acyl_, whereas the efficiency of the pong step is reflected by *k*_cat_/K′_mal_. 

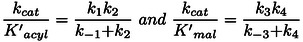




As depicted in Figure 3C–D, enzymatic efficiencies of the KS increase with chain length in both the ping and pong steps. According to equation 2, an increased *k*_cat_/K′ can have various origins, including one or more of the following: **a**) an increased KS affinity towards the substrate and, therefore, an equilibrium shift towards the enzyme‐substrate complex (increased k_1_ or k_3_ over k_−1_ or k_−3_ respectively), **b**) an increased reaction rate (increased k_2_ or k_4_) or **c**) an increased dissociation of the enzyme‐product complex (increased k_2_ or k_4_).

In the case of the ping step, a change in the dissociation of the enzyme‐product complex (**c**) is unlikely, as, after the substrate is delivered to the KS, the unloaded holo‐ACP will most likely have the same affinity towards the acyl KS, regardless of the identity of the KS‐bound substrate. Therefore, the increase in the enzymatic efficiency of the ping step with acyl chain length is likely due to either the affinity of the KS towards the acyl‐ACP (**a**) or the transacylation rate (**b**). In the case of the pong step, we do not expect an impact of the acyl chain resting in the KS on the affinity towards malonyl‐ACP (**a**). Assessing the impact of chain length on the decarboxylation and bond formation processes is significantly more challenging (**b**). Mutational studies have shown the putative influence of a bound acyl chain to the decarboxylation.^[38]^ On the other hand, the differences in chain length are relatively distant from the malonyl binding site, making a significant influence on the decarboxylation reaction seem unlikely. Therefore, the improvement in the enzymatic efficiency of the pong step with chain length is likely to reflect increases in the rate of condensation (**b**) or the dissociation of the enzyme‐product complex (**c**). Based on these considerations, we expected that the acyl chain lengths would have a more significant influence on the ping step than on the pong step of the KS mediated reactions. This assumption is well reflected by the more extensive shift of the ping enzymatic efficiency depending on the acyl chain length (Figure 3C–D). We conclude that the ping step, the transacylation reaction that delivers the acyl moiety to the KS, is the determining factor in the (chain length) specificity of the KS.

Our data reveals the chain length dependency of the enzymatic efficiency as well as its components *k*_cat_ and K′ (Figure 3C–G). The enzymatic efficiency *k*_cat_/K′_acyl_ increases with chain lengths from two to ten carbon atoms (2.6 ± 0.8 mM^−1^s^−1^ for C2, 24 ± 3 mM^−1^s^−1^ for C6 and 53 ± 6 mM^−1^s^−1^ for C10; Figure 3C). Increasing enzyme activity with increasing chain length from very short to medium acyl chains for the KS catalyzed transacylation reaction was observed before when employing acyl‐CoA substrates and monitoring CoA release.[^25^, ^37^] The enzymatic efficiency *k*_cat_/K′_acyl_ determined here is 1–2 orders of magnitude larger than determined by the transacylation assay previously, which we assume to originate from employing acyl‐ACPs as the native substrates. However, despite the divergent absolute values, the trends in the transacylation part‐reaction and the overall KS mediated reaction are identical, substantiating the finding that substrate specificity and cooperativity originate from the ping step.

The enzymatic efficiency of the mFAS‐KS is approximately 2–14 times higher than that of the homologous type II proteins KasA and KasB of *M. tuberculosis* and FabF and FabB of *E. coli* (Figure S13).[^47^, ^56^] However, values for turnover number *k*_cat_ and *K*_m,acyl_ (K′_acyl_) vary individually more substantially between mFAS‐KS and type II proteins. Notably, *k*_cat_ of mFAS‐KS is 29–110 times higher compared to these bacterial enzymes, corresponding to faster catalysis, and K′_acyl_ values are 5–43 times higher than the *K*_m_ of the type II enzymes, indicating lower affinity. We suggest that the large K′_acyl_ of mFAS‐KS is a result of the high local concentration of the substrate due to its covalent linkage to the ACP domain. The high K′ ensures transient binding of the ACP to the KS, which is necessary to facilitate rapid docking to all domains in the FAS complex.

The turnover number of the full‐length mFAS, measured by NADPH consumption of the internal keto reductase and enoyl reductase domains was previously determined to be 3.1 ± 0.3 s^−1^ (Figure S13).^[32]^ As an approximation, this overall turnover number corresponds to the average of the turnover numbers of all chain lengths. We have recorded similar rates for KS with the substrate C10‐ACP only (3.2 ± 0.2 s^−1^; rates for other chain lengths are slower), such that the KS assay seems to underestimate KS elongation rates. Differences between the KS rates in the native enzyme complex (ACP integrated into the polypeptide) and the assay set up (stand‐alone ACP) might have their origin in factors such as molecular steering. This caveat aside, the MabA assay accurately reflects the kinetics of the integrated KS.

### Analysis of the Acyl Binding as the Origin of Cooperativity

**Figure 4:**
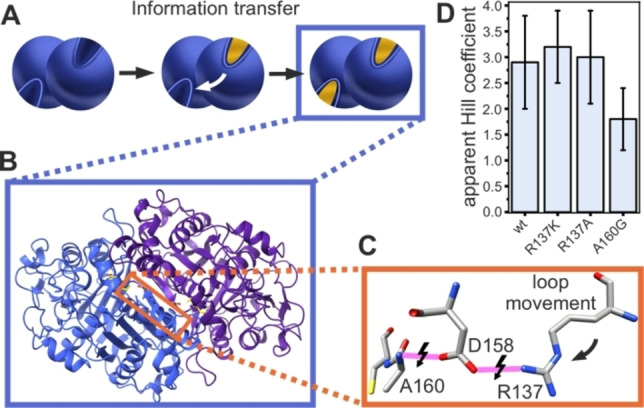
Proposed hydrogen bond network. **A** A simplistic Scheme illustrating the principle of classical positive cooperativity. The binding of a substrate to one active site leads to an increased affinity of the second active site towards the same substrate. **B** The KS structure in cartoon display shows the position of the hydrogen bond network (marked in orange). The two protomers are colored in blue and violet, respectively, and the bound substrate octanoyl is colored in yellow. **C** The hydrogen bond network of R137, D158 and A160. Hydrogen bonds are colored in pink and may change depending on binding site occupancy. **D** Apparent Hill coefficient of the active variants. Bars heights represent the means of three biological replicates and the black error bars standard deviation.

As discussed in the preceding section, KS activity shows a sigmoidal dependence on acyl‐ACP concentration. The sigmoidal shape indicates a positive cooperative working mode of the dimeric KS domain, which essentially means that substrate turnover by one protomer promotes turnover by the other protomer (Figure 4A). For cooperativity, information about the catalytic status of protomers needs to be exchanged. In a previous work, a hydrogen bond network was proposed to enable protomer communication (Figure 4B–C):^[37]^ Upon comparing crystal structures of the KS‐MAT didomain in the unbound (apo) and octanoyl‐bound states, a conformational change in active site residues was observed. Morphing these states suggests that during octanoyl binding the gatekeeping residue F395.A (chain A) rotates and induces a loop to shift in the other protomer harboring residues M132.B, Q136.B and M139.B (chain B). The loop displacement causes a conformational change of R137.B that is in interaction with binding site residues D158.B and A160.B through a hydrogen bond network (Figure 4C). To evaluate whether these coordinated changes are the molecular basis for cooperativity, amino acids of this hydrogen bond network were mutated. The steepest sigmoidal response curve of the wildtype was measured with C10‐ACP; hence, enzyme variants were evaluated by titration of C10‐ACP (Figure S14, Table S2). For variants D158 N, D158S and A160 V, activity was abolished. As the protein quality seemed to be intact given the maintained dimeric state (Figure S15), the reasons for inactivity may be the inability of substrate binding or processing. Variants R137 K and R137 A do not seem to alter the apparent Hill coefficient (3.2 and 3.0, respectively, compared to 2.9 of wildtype), which indicates that the residue R137 is not participating in protomer communication. We observed a slight impact of the A160G variant on the cooperativity, although not statistically significant based on ANOVA‐test results (see SI) (Figure 4D). In conclusion, this mutational study shows, that the proposed H‐bond network does not provide a mechanistic explanation to the cooperative kinetics.

### Analysis of Carrier Binding as the Origin of Cooperativity

**Figure 5:**
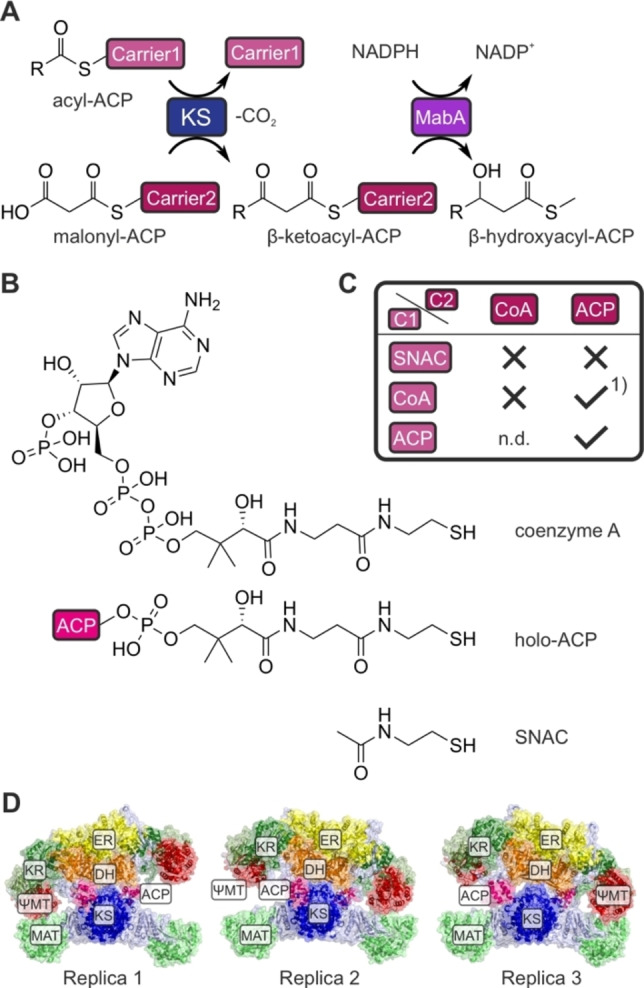
Analysis of the impact of the carrier in cooperativity. **A** Scheme of the MabA assay with two different carriers. The acyl‐starter substrate is bound to carrier 1 and the malonyl extender substrate is bound to carrier 2. **B** In the MabA assay, various carrier types can be applied, including N‐acetylcysteamine (SNAC), coenzyme A (CoA) and ACP. All these carrier types share the N‐acetylcysteamine group, whereas the CoA and ACP carriers additionally share the Ppant moiety. **C** Combination of carrier of starter and extender substrates changes KS kinetics. In the table, positive cooperativity is marked with a tick, whereas the absence of a sigmoidal response is marked with an X. 1) The analysis was performed with holo‐ACP as the second substrate during transacylation measurement by Rittner et al.^[37]^ **D** The rat fatty acid synthase (rFAS) structure after the first, second and third 400 ns MD replicas. The simulation time of replica 1 was extended to 1 μs afterward. The alphafold structure served as a template (AF−P12785‐F1). A rigid‐body rearrangement of the two wings in rFAS promoted a conformation with slightly shorter and longer linkers in all MD simulations. The two ACP domains remained bound to the KS domains during the whole MD simulations in two of the three replicas. On the third replica, the ACP domains dissociated from the KS domains after a conformational shift. This behavior is expected and aligns with the well‐known transient nature of the ACP‐KS association. The ACP‐KS interface was constituted by residues 45–49, 198–205, 297–298 (KS), and 47–74 (ACP), with an area of 197 Å^2^.

The apparent Hill coefficients of the wildtype and the R137 mutants are higher than expected for a dimeric protein, which should exhibit a Hill coefficient of two or lower, showing the minimal number of binding sites.[^57^, ^58^] This indicates that the interpretation of the sigmoidal‐shaped kinetics of the KS‐mediated reaction as positively cooperative only taking the acyl moiety of the starting substrate into account does not capture the full complexity of the condensation reaction. In seeking to reveal the role of the substrate for KS cooperativity, we performed the coupled assay with substrates that are systematically altered in the carrier part and analyzed whether sigmoidal profiles in velocity vs. substrate concentration plots were retained (Figure 5A). In nature, the KS substrates are tethered to the thiol moiety of a post‐translationally introduced phosphopantetheine (Ppant) arm of ACP (Figure 1). Homologue versions of acyl‐ACP are acyl‐CoA and acyl‐SNAC (Figure 5B): The larger CoA captures the interaction of Ppant with the binding tunnel that may facilitate correct binding of the substrates. As coenzyme A contains an additional adenosine‐3’,5’‐diphosphate, not present in acyl‐ACP, additional attractive or repulsive interactions may be induced. The SNAC moiety is a mimic of the distal end of the Ppant and therefore represents a truncated acyl‐ACP.

Those carriers were used in the MabA assay, both as part of starter‐carrier (donor) and extender‐carrier (acceptor) substrates. Based on three datasets, it was found that cooperativity does not simply stem from the acyl moiety, but its origin is more complex: Dataset 1, published previously by our lab:^[37]^ The use of hexanoyl‐CoA (C6‐CoA) as the donor substrate and holo‐ACP as the acceptor substrate in the transacylation assay results in a sigmoidal shape of the collected data. Dataset 2: The use of C6‐SNAC (instead of C6‐CoA, dataset 1) and Mal‐ACP in the MabA assay results in the loss of the sigmoidal shape (Figure 5C, S8). Dataset 3: The use of C6‐CoA and Mal‐CoA in the MabA assay also results in the loss of the sigmoidal shape (Figure 5C, S8). From datasets 1 and 2, it can be concluded that cooperativity emerges already in the transacylation reaction (PING step), and that cooperativity requires additional interactions provided by the Ppant arm (SNAC alone is not sufficient to induce cooperativity). Dataset 3 indicates that ACP in the PONG step of the reaction cascade is contributing to the cooperativity. The cooperativity of the KS involving both starter‐ and extender‐substrate could facilitate cross‐synchronization of the PING (first chamber) and PONG step (second chamber) (Figure S16).

Our findings are in accordance with the transacylation analysis of rat FAS, which utilized CoA and pantetheine as carriers and did not reveal any signs of cooperativity.^[25]^ It is further noted that the impact of (Mal−)ACP on the cooperative response was already seen in fluorometric binding assays of the KS FabF from *E. coli*.^[59]^

Since the structural prerequisite for cooperativity in the KS mediated reactions is the simultaneous binding of ACP domains, we next asked how the mFAS scaffold inherently allows the binding of the ACP domains to both protomers of the KS dimer simultaneously.^[60]^ We performed molecular docking and MD simulations, which confirmed that the linker of 10 amino acids (V2104‐H2113), through which ACP is attached to KR, ensures sufficient conformational flexibility for simultaneous binding to both KS dimers (Figure 5D). The simulations further show a rigid‐body conformational variability of the condensing and modifying wing leading to different distances of the KR and KS domains (Table S3), while no considerable changes in domain structures were observed (Figure S17–19). The rigid movement of the condensing and modifying wing was observed previously. The rearrangement within the modifying wing found in the cited study could not be corroborated by our simulation.^[19]^ This overall conformation, which has the ACP docked at the KS in both protomers and a reduced angle between the two wings of the mFAS, serves as a picture of the mFAS‐complex during the KS‐catalyzed reaction in fatty acid synthesis. We would like to point out that previous data on heterodimeric mFAS, in which only one of two KS‐domains was knocked out in function by mutation, can be captured via the cooperative properties: Here, the transacylation activity of the KS domain recorded with a non‐cooperative (non‐ACP) carrier reached expected 50 %, while full fatty acid synthesis (ACP as carrier) did not reach 50 % activity.^[61]^ These results further imply that the cooperativity of the KS dimer has an influence on the native fatty acid synthesis in the mFAS‐complex.

We note that our data cannot distinguish whether cooperativity results from a sequential or a concerted process; therefore, it remains unclear whether the symmetry model by Monod‐Wyman‐Changeux or the sequential model by Koshland‐Némethy‐Filmer is the appropriate theoretical foundation for describing KS cooperativity.

### Monitoring Intermediates of the Fatty Acid Cycle

**Figure 6:**
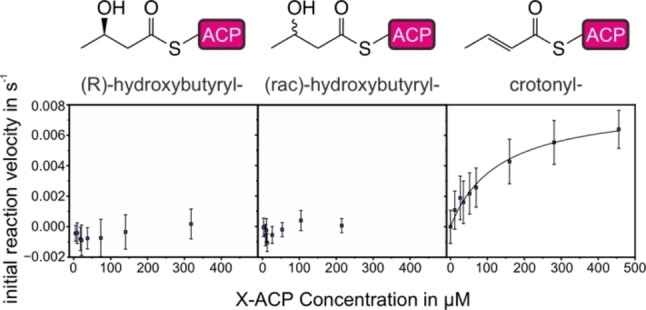
Kinetics of intermediates of the fatty acid cycle. Titration of (*R)‐*HB‐ACP, (*rac*)‐HB‐ACP and crotonyl‐ACP. Measurements were performed with 1.5 μM KS and 70 μM Mal‐ACP. All data points represent the means of three biological replicates, and the error bars represent the standard deviations.

The metazoan FAS produces saturated fatty acids with high specificity, which indicates that the KS selects for saturated acyl‐moieties, which are produced by the ER‐mediated reduction. However, to the best of our knowledge, quantitative kinetic data on the selectivity of the condensation reaction is elusive. With the MabA‐assay in hand, we sought to analyze the selectivity of the KS, particularly for preferring fully reduced substrates over intermediates of the fatty acid cycle. Intriguingly, previous work has shown that the mFAS is able to produce triacetic acid lactone (TAL) under non‐reducing conditions at condensation rates of 16 % of fatty acid production, which proves that the KS is, in general, able to condense β‐keto acyl‐units.^[32]^ To analyze KS selectivity, the fatty acid cycle intermediates (*R*)‐β‐hydroxybutyryl‐ACP ((*R)‐*HB‐ACP) and crotonyl‐ACP were applied in the MabA assay (Figure 6). The β‐ketoacyl intermediate, the product of the KS‐mediated elongation, is a substrate of MabA and was therefore not part of this study.

Conducting the assay with (*R)‐*HB‐ACP, a representative second intermediate, did not result in detectable activity (Figure 6), suggesting that the KS is unable to condense β‐hydroxyacyl‐ACP and Mal‐ACP. To rule out a false negative result caused by MabA failing to reduce the condensation product in the coupled reaction, the reaction mixture was additionally analyzed by urea PAGE (Figure S20). In case the KS accepts (*R)‐*HB‐ACP for condensation with Mal‐ACP, both substrates would be consumed during the time course of the reaction. Urea PAGE shows that the amounts of Mal‐ACP and HB‐ACP remain constant over one hour of reaction, confirming that (*R)‐*HB‐ACP is not a substrate for KS. We also considered whether the KS may just counterselect against elongation of the (*R)‐*HB‐ACP enantiomer, which natively occurs as an intermediate in the fatty acid cycle but not against (*S)‐*HB‐ACP. However, use of racemic HB‐ACP to the MabA assay, did not result in any observable activity. (Figure 6).

For crotonyl‐ACP, the third fatty acid cycle intermediate, we observed KS activity that was lower compared to saturated fatty acids (k_cat_^app^=81 ± 5 x10^−4^ s^−1^ and K_m_^app^=0.13 ± 0.02 mM at 70 μM Mal‐ACP). Thus, discrimination of the FAS against unsaturated fatty acyl moieties (Figure 6) is less pronounced than for the β‐hydroxy compounds. The specificity of the KS towards acetyl‐ACP compared to crotonyl‐ACP is (*k*_cat_/K′_acetyl_)/(*k*_cat_/*K*_m,crot_)=42. According to the Transition State Theory, the transition state energy difference that leads to a difference in specificity is ΔΔG=9 kJ/mol, which equals less energy than a weak hydrogen bond.

In conclusion, this data shows that the KS gatekeeps fatty acid synthesis by selecting against fatty acid cycle intermediates, thereby achieving product fidelity. The data also indicates that the discrimination of KS against the intermediates of the fatty acid cycle arises due to the different functionalization of the acyl moiety, with a double bond (assay substrate crotonyl‐ACP) allowing less pronounced discrimination than a hydroxyl group (assay substrate HB‐ACP).

### Computational Analysis of Substrate Selectivity by KS

**Figure 7:**
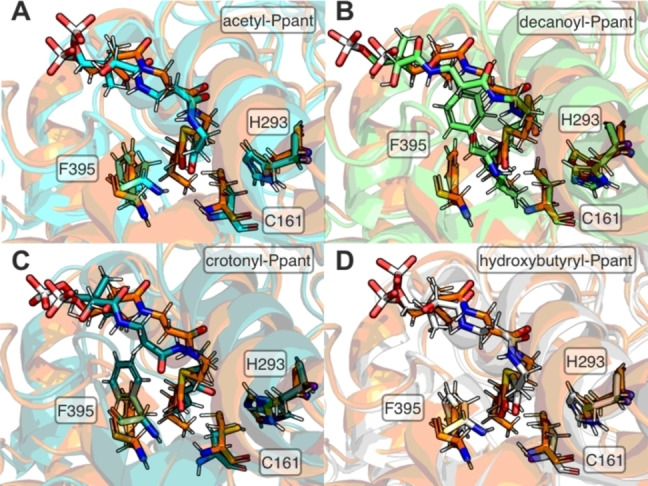
Snapshots from the MD Simulation of acyl‐Ppant substrates in the KS active site. **A** Structure of hexanoyl‐Ppant (orange) superimposed to acetyl‐Ppant (cyan) and the catalytic relevant residues C161, H293, F395. **B** Superposition of hexanoyl‐Ppant (orange) and decanoyl‐Ppant (green). **C** Superposition of hexanoyl‐Ppant (orange) and crotonyl‐Ppant (teal). **D** Superposition of hexanoyl‐Ppant (orange) and hydroxybutyryl‐Ppant (silver).

Our experimental data shows that the transacylation (ping) step can account for substrate specificity of the KS‐mediated reaction (ping‐pong, Figure 1A), which aligns with previous data monitoring the isolated transacylation kinetics.^[37]^ To obtain an atomic‐level understanding of the specificity of the reaction, we performed MD simulations on the KS:acyl‐Ppant complex formed during transacylation. Specifically, we compared the stability of acetyl, hexanoyl, decanoyl, crotonyl, and (R)‐hydroxybutyryl substrates within the active site of the KS (Figure 7). The RMSD analysis of the protein backbone revealed the overall stability of KS throughout the 100 ns simulations (Figure S21–S22). An analysis of the interatomic distances between the conserved amino acids Cys161, His293 and Phe395, which are highly correlated with enzyme‐substrate reactivity was carried out for each simulation (Table 1). Their role is in brief: Cys161 is deprotonated in the first step of the catalytic mechanism. The deprotonation involves His293 as suggested by in silico studies,^[62]^ although alternative mechanistic models have also been proposed.^[38]^ The deprotonation is accompanied, in a probably concerted step, by a nucleophilic attack on the Ppant‐substrate thioester carbon by the Cys161 thiolate. Finally, Phe395 together with Cys161 forms an oxyanion hole stabilizing the negative charge at the carbonyl oxygen generated in the transition state. The latter effect lowers the activation free energy of 18.8 kJ/mol at most.^[63]^ The distances of the four described interactions were analyzed (Figure S23).

**Table 1:** Average distances and standard deviations for important catalytic interactions to the first step of the reaction mechanism. All distances are presented in Ångström. Additionally, the sum of catalytic and oxyanion hole distances was computed to facilitate its interpretation. If the solvent deprotonates Cys161, distance d1 should be excluded from the analysis. Under this assumption, the catalytic and oxyanion distances account for the lack of reactivity in crotonyl, but not in (R)‐HB.

	Catalytic Distances (Å)			Oxyanion Hole Distances (Å)		
	**C161(SH)‐** **H293(Nϵ)** **(d1)**	**C161(S)‐** **Ppant(C)** **(d2)**	**Sum of the** **catalytic** **distances**	**C161(HN)‐** **Substrate(O)** **(d3)**	**F395(HN)‐** **Substrate(O)** **(d4)**	**Sum of the** **oxyanion hole** **distances**
Acetyl	5.2±0.2	3.3±0.2	8.5	2.1±0.1	3.1±0.3	5.2
Hexanoyl	4.9±0.6	3.5±0.2	8.4	2.9±0.5	3.9±1.2	6.8
Decanoyl	4.7±0.9	3.6±0.4	8.3	3.9±1.5	2.9±1.3	6.8
Crotonyl	4.5±1.1	4.3±1.1	8.8	4.3±1.3	5±2	9.3
(R)‐HB	5.9±1.0	3.3±0.2	9.2	2.9±0.3	2.1±0.2	5.0

Acetyl, hexanoyl, and decanoyl display low distances between catalytic residues (Table 1, Figure 7A–B), except for the Cys161‐SH—His293‐Nϵ distance in acetyl‐Ppant, known to be the least reactive of the three. Shorter oxyanion hole hydrogen bond lengths partially compensate for this for the latter. This data indicates that the reactivity of the transacylation complex affects the enzymatic efficiency and, thus, specificity. However, the data cannot rule out that the affinity of acyl‐Ppant to KS is increased (an increase of k_1_ over k_−1_, see equation 2) and contributes to raising enzymatic efficiency.

Upon examination of the interatomic distances, it became evident that crotonyl displays higher, less favorable distances between catalytic residues and longer oxyanion hole hydrogen bonds (Figure 7C), consistent with its lower reactivity within the active site of KS. R‐HB, on the other hand, seems to be suitably aligned in the active site, given its close distances to Cys161‐SH and to the oxyanion hole forming backbone amide groups (Table 1, Figure 7D). Still, the distance of the hydrogen bond between the Cys161‐SH and the His293‐Nϵ is high, precluding the Cys161 deprotonation and subsequent nucleophilic attack on the Ppant (Table 1).

In general, the study reveals that the experimentally measured enzymatic efficiencies of all substrates are linked to the tightness of catalytic residue interactions at the reactant state, represented by their interaction distances. In addition, the study suggests that the transacylation kinetic is mostly correlated with the easiness of Cys161 deprotonation and nucleophilic attack on Ppant thioester carbon and, on a lesser scale, the stabilization of the negative charge accumulated at the Ppant thiolate by the oxyanion hydrogen bonds. Here, we refer once again to the role of His293 in deprotonation, as suggested by Lee and Engels through computational approaches.^[62]^ However, the origin of the deprotonation is much more controversial. Of particular significance is data showing that the mutation of His293 to alanine, does not decrease (but even increase) reaction rates, leading to a model in which Cys161 is prone to deprotonation by a helix dipole effect.^[38]^

## Conclusion

The FAS multienzymes are crucial for the synthesis of long‐chain fatty acids from acetyl‐CoA and malonyl‐CoA through a series of enzymatic reactions. Herein, the KS domain catalyzes the central carbon‐carbon bond forming reaction and thus holds key responsibility for the fidelity of long‐chain fatty acid synthesis. So far, the enzyme kinetic analysis of mFAS catalytic domains were limited by using substrate analogs acyl‐Ppant or acyl‐CoA, resulting in properties of the reactions not being observable.[^25^, ^29^, ^31^] Here, for the first time, we paint the full picture of the KS catalysis using an enzyme‐coupled assay with close‐to‐nature kinetics. Our analysis reveals that the KS catalysis is dependent on the chain length of the fatty acid intermediate. Specifically, it exhibits increasing substrate specificity from short to medium saturated fatty acids, which unveils that the first cycle (condensation of acetyl‐ and malonyl moieties) is the slowest in fatty acid synthesis. The elongation rates of the KS correlate well with the rates of the multienzyme, which indicates that the KS is rate‐limiting for the fatty acid biosynthesis.

The KS kinetic properties are particularly interesting when considered in the context of the MAT domain, which loads and unloads ACP domains with acyl groups (Figure 1A).[^24^, ^25^, ^27^] The relatively slow elongation of short acyl chains by the KS is in contrast with the very rapid transacylation of these compounds by the MAT. For the overall biosynthesis, this means that short acyl chains bound to FAS will exhibit high tendency to be unloaded by the MAT. In this function, the MAT depends on the concentration of acetyl‐CoA and malonyl‐CoA as substrates, as they compete for the MAT binding pocket. The concentration of malonyl‐CoA has been described to vary depending on the metabolic state of a cell,^[64]^ such that offloading of short acyl chains will be promoted at low concentrations, while at higher malonyl‐CoA concentrations FAS will overcome the kinetic lag phase imposed by KS. Thus, via the interplay of KS and MAT, FAS is offered direct feedback on the metabolic state of the cell.

The titration of substrates revealed a positive cooperative response in KS activity, which was further investigated. We determined Hill coefficients for substrates with different chain lengths and bound to different carriers (ACP, CoA and SNAC), as well as of KS mutants, in order to modify the hydrogen bond network that bridges the protomers of the KS dimer. Our data suggests that the ACP plays a crucial role in triggering the cooperative response of the KS. Although the molecular basis for cooperativity was not fully elucidated, we speculate that cooperativity, which increases from short to medium chain length, contributes to increasing enzymatic efficiency of the mFAS by synchronizing the elongation reaction in the two reactions clefts and confining the higher‐order conformational dynamics of mFAS, that is the positional variability of the domains within the multidomain complex. The metazoan FAS exists as an ensemble of interconverting conformational states, of which a state in which both ACPs engage with the KS dimer is more likely to be found for a cooperative KS than for an uncooperative KS.

Further, this study showed that the fidelity of the mFAS derives from the discrimination of the KS against fatty acid cycle intermediates (tested with HB‐ACP and crotonyl‐ACP). This discrimination was demonstrated with the enzyme‐coupled assay. MD simulations showed elongated, unproductive catalytic distances of the crotonyl‐Ppant and HB‐Ppant complexes compared to that of saturated intermediates. The selectivity of the reaction of the KS emerges from the first step (ping step) of the double displacement reaction mechanism. This is plausible, as the sequence of steps in KS‐mediated condensation is reversible up to the loading into the KS (before decarboxylating Claisen condensation), thus allowing correction of false KS loading without incurring energetic costs.

As shown by the kinetic measurements, the presented MabA assay is suitable for monitoring reaction kinetics and enzyme‐specific properties. As the KS is the most conserved domain in the genetically closely related megasynthases FAS and PKS across species, it is likely that the same method can be applied to KS from several other systems, including PKSs. The present study thus contributes to a deeper understanding of the reaction sequence, domain interactions and specificities in type I multienzymes. By offering access to the enzyme kinetic details of the KS and its mutants, it will facilitate protein engineering approaches that, in turn, can grant access to new‐to‐nature biosynthetic pathways. In addition, the details about KS specificity can provide valuable insights for the design of new FAS‐inhibition therapeutics for the treatment of obesity and cancer.

## Supporting Information

The authors have cited additional references within the Supporting Information.[^65^, ^66^, ^67^, ^68^, ^69^, ^70^, ^71^, ^72^, ^73^, ^74^, ^75^, ^76^]

## Conflict of Interests

The authors declare no conflict of interest.

1

## Supporting information

As a service to our authors and readers, this journal provides supporting information supplied by the authors. Such materials are peer reviewed and may be re‐organized for online delivery, but are not copy‐edited or typeset. Technical support issues arising from supporting information (other than missing files) should be addressed to the authors.

Supporting Information

Supporting Information

Supporting Information

Supporting Information

Supporting Information

Supporting Information

## Data Availability

The data that support the findings of this study are available from the corresponding author upon reasonable request.
