# Supplementary material for: The Kinetics of Carbon‐Carbon Bond Formation in Metazoan Fatty Acid Synthase and Its Impact on Product Fidelity
Source: Angew Chem Int Ed Engl. 2024 Dec 4;64(2):e202412195. doi: 10.1002/anie.202412195 (PMC11720392; doi:10.1002/anie.202412195)
Supplement: Supplementary file 6 — Supporting Information [file ANIE-64-e202412195-s006.pdf]

Angewandte Chemie
Eine Zeitschrift der Gesellschaft Deutscher Chemiker

Supporting Information

The Kinetics of Carbon-Carbon Bond Formation in Metazoan Fatty Acid Synthase and Its Impact on Product Fidelity

*C. Gusenda, A. R. Calixto, J. R. Da Silva, P. A. Fernandes, M. Grininger**

Supporting Information

The Kinetics of Carbon-Carbon Bond Formation in Metazoan Fatty Acid Synthase and Its Impact on Product Fidelity

Christian Gusenda[a], Ana R. Calixto[b], Joana R. da Silva[b], Pedro A. Fernandes[b] and Martin Grininger*[a]

[a] C. Gusenda, Prof. M. Grininger
Institute of Organic Chemistry and Chemical Biology
Buchmann Institute of Molecular Life Sciences, Goethe University Frankfurt
Max-von-Laue-Str. 15, 60438 Frankfurt am Main, Germany
E-mail: grininger@chemie.uni-frankfurt.de

[b] A.R. Calixto, J.R. da Silva, Prof. P.A. Fernandes
LAQV, REQUIMTE, Departamento de Química e Bioquímica,
Faculdade de Ciências, Universidade do Porto
Rua do Campo Alegre s/n, 4169-007 Porto, Portugal

Additional Materials and Methods

Cloning. Plasmids of MabA, Sfp, ACP and KS-MAT were readily available as stated in the section "sequences" of this SI. The point mutations for cooperativity studies were introduced pcr based with primers mentioned in the section "primers". CloneAmp^{TM} HiFi PCR Premix (Takara) was used, and PCR mixtures were prepared according to manufacturer's guidelines. The initial denaturation of the template KS-MAT^{S581A} was run at 98°C for 180 s, followed by 23 cycles of 98°C for 10 s, 75°C for 20 s and 72°C for 90 s. The final elongation was performed at 72°C for 420 s. The template was digested with DpnI (NEB) at 37°C for 1 h. 1 µL of PCR-Mix was transformed into 40 µL of Stellar^{TM} competent cells (Takara) and grown in LB-Agar (containing 100 µg/mL Amp and 1% glucose). One clone each was used to inoculate 8 mL of LB medium, which was incubated at 200 rpm and 37°C in culture tubes O/N. The plasmid was extracted from the cells using GeneJET Plasmid Miniprep Kit (Thermo Scientific) following manufacturer's instructions.

Plasmid transformation. To transfer the plasmid encoding for the desired protein, 40 µL BL21 gold *E. coli* cells (Novagen) (F-, ompT, hsdSB(rB- mB-), dcm+, Tetr, galλ (DE3) endA, Hte) were pipetted to 1 µL plasmid solution. The mixture was kept on ice for 15 min prior to a 20 sec heat-shock at 42°C. The cells were again kept on ice for about 5 min. After adding 400 µL super optimal medium with catabolite repression (SOC), the mixture was incubated for 1 h at 37°C. After the incubation, the cells were centrifuged at 800 x g for 3 min. 340 µL of the supernatant was discarded and the residual 100 µL of resuspended cells were plated onto LB agar plates (containing 100 µg/mL Amp and 1% glucose) using glass beads. The plates were incubated at 37°C over night (O/N).

Protein expression. To prepare a preculture, a single clone (in case of plasmid preparation) or 5 clones (in case of protein expression) was transferred to 20 mL of LB medium (containing 100 µg/mL Amp and 1% glucose) and incubated at 37°C O/N. The preculture was transferred into 1 L of TB medium (containing 100 µg/mL Amp) as main culture and incubated at 37°C until an optical density (OD_{600}) of 0.6 to 0.8 was reached. The main culture was cooled down and the expression was induced by adding 250 µL isopropyl β-d-1 thiogalactopyranoside (IPTG). The proteins were expressed at 20°C O/N. After about 16 h the main culture was transferred to centrifuge tubes from Beckmann Coulter and centrifuged in the Beckmann Coulter Avanti J20-XP with rotor JLA-8.100 at 4500 x g for 20 min. The supernatant was discarded, and the cells were resuspended in 20 mL His-wash buffer (200 mM KCl, 50 mM potassium phosphate, 30 mM imidazole, 10% glycerol, pH 7.0). To the suspension a small amount of Dnase I (deoxyribonuclease) (Sigma Aldrich) and 1 mM ethylenediaminetetraacetic acid (EDTA) was added. The cells were disrupted using the French Pressure Cell Press in one cycle. The obtained mixture was centrifuged using the JA 25.500 rotor at 40000 x g for 1 h. The supernatant was collected.

Protein purification. The crude extract was transferred to a His-NTA column (Takara) and washed with 5 CV (15 mL) of His-wash buffer. The protein was then eluted two times with 2.5 CV (7.5 mL) His-elution buffer (200 mM KCl, 50 mM potassium phosphate, 300 mM imidazole, 10% glycerol, pH 7.0) and transferred to a Strep-Tactin column. The column was washed with 2 CV (10 mL) Strep-wash buffer (250 mM potassium phosphate, 1 mM EDTA, 10% glycerol, pH 7.0). The protein was finally two times eluted with 3 CV (15 mL) Step-elution buffer (250 mM potassium phosphate, 1 mM EDTA, 10% glycerol, 2.5 mM desthiobiothin, pH 7.0). The obtained protein solution was concentrated and stored at -80°C until further purification. Proteins were further purified and analyzed after tandem affinity chromatography by SEC using the ÄKTA Basic system (GE Healthcare). The eluent was filtered, degassed and cooled before usage. The KS-MAT proteins were incubated for 1 h at 37°C prior to SEC to

support dimerization. The protein sample was filtered using Ultrafree® Durapore® centrifugal filters (Merck). The KS-MAT constructs were purified using the Superdex 200 10/300 GL (cytiva) and Strep-wash buffer as eluent, whereas the ACP, MabA and Sfp proteins were purified using the HiLoad 16/600 Superdex 200 (cytiva) and ACP-SE buffer (50 mM potassium phosphate, 200 mM KCl, 10% glycerol, 1 mM EDTA, pH 7.0), MabA-SE buffer (50 mM sodium phosphate, 450 mM NaCl, 20% glycerol, pH 7.5) and Sfp-SE buffer (50 mM HEPES, 250 mM NaCl, 2 mM $MgCl_2$, 10% glycerol, pH 8.0) as eluent respectively. The column was equilibrated with 2 CV of buffer prior to usage. The protein sample was injected with automatic rinsing with two times the loop volume and eluted by 1.2 CV of buffer. All other settings were adjusted according to the manual.

Acyl-CoA synthesis. This synthesis was adopted from Peter et al. and Valenzano et al.[67,68] The respective acid (0.31 mmol, 6 eq.) was dissolved in 2 mL THF and cooled to 0°C. 40 µL triethylamine (0.29 mmol, 6 eq.) and 27 µL chloroformate (0.31 mmol, 6 eq.) were pipetted to the solution and stirred for 45 min at 0°C under argon. The reaction mixture was transferred to a 2 mL microcentrifuge-tube and centrifuged for 5 min at 20000 x g to get rid of insoluble salts. The supernatant was transferred to 40 mg CoA (49 µmol, 1 eq) in 2 mL 0.1 M sodium hydrogen carbonate solution. The mixture was stirred for 1 h at room temperature. The reaction mixture was poured into 25 mL of cold acetone (-20°C) and centrifuged for 5 min at 10000 x g. The raw products were obtained as colorless solids and stored at -20 °C until further purification. To isolate the CoA-esters, the samples were dissolved in 200 mM ammonium acetate at pH 6 and centrifuged for 2 min at 20000 x g to separate any solids. The product solution was injected to the Acclaim. Polar Advantage II LC-Column (Thermo Scientific) and eluted with a buffer (200 mM ammonium acetate, pH 6)-acetonitrile gradient (10 - 60% acetonitrile). The combined product fractions were concentrated under vacuum. The product was obtained at a solution in buffer. The yield was determined by measuring the concentration of the product solution. The products were analyzed using ESI-MS and HPLC. Acetyl-CoA: 13 nmol, 26%, *m*/*z* $[M+H]^+$ = 810.40 / Butyryl-CoA: 13 nmol, 26%, *m*/*z* $[M+H]^+$ = 838.08 / Hexanoyl-CoA: 16 nmol, 33%, *m*/*z* $[M+H]^+$ = 866.20 / Octanoyl: 18 nmol, 36%, *m*/*z* $[M+H]^+$ = 894.19 / Decanoyl-CoA: 15 nmol, 31%, *m*/*z* $[M+H]^+$ = 922.22.

Acyl-ACP Synthesis. To functionalize purified apo-ACP with acyl-moieties a Sfp phosphopantetheinylation was performed. 3 mM acyl-CoA, 600 µM apo-ACP and 30 µM Sfp in Sfp-reaction buffer (50 mM HEPES, 200 mM NaCl, 10 mM $MgCl_2$, pH 7.0) was incubated at 37°C for 20 min. (R)-Hydroxybutyryl-ACP was synthesized in respective solution with acetoacetyl-CoA (AA-CoA) and additional 30 µM MabA and 6 mM NADPH. To separate the product acyl-ACP from Sfp and acyl-ACP, Strep tactin affinity chromatography was performed. The solution was pipetted onto 5 mL of Strep-Tactin®XT. 4Flow® resin. The resin was subsequently washed with 3 CV Strep-wash buffer and acyl-ACP was eluted with 2.5 CV StrepXT-elution buffer (250 mM potassium phosphate, 1 mM EDTA, 10% glycerol, 50 mM biotin, pH 7.0). The elution fraction was rebuffered in ACP-buffer by using Amicon® centrifugal filters (Merck) by diluting the sample with ACP-buffer and concentrating consecutively until a final concentration of 1 % of the original buffer was reached. The fractions were analyzed with HPLC or urea PAGE (figure S2-S3). The analysis of different ACP species was carried out with HPLC on a Discovery®BIO Wide Pore C5 (Merck) with 0.1% TFA in water and 0.1% TFA in acetonitrile (39-42% CH_3CN in 12 min with C2, C4 and crotonyl-ACP; 20-80% CH_3CN in 15 min with C6 - C14-ACP). Around 0.25 nmol sample was injected to the column.

Urea PAGE analysis. Analysis of malonyl-ACP and hydroxybutyryl-ACP was performed with urea PAGE. Adapted from literature, the gel electrophoresis was carried out in a polyacrylamide gel containing urea.[65,66] The separating gel contained 15% acrylaminde/ bisacrylamide, 1.12 M Tris, 7.5 M urea, 0.1% (v/v) N,N,N',N'-tetramethylethyldiamine and 0.03% (w/v) ammonium persulfate. The loading dye (2x) contained 1% bromphenol blue, 25%

glycerol, 62.5 mM Tris, 2 M urea and either 10 mM N-ethylmaleimide (NEM) or N-aminoethylmaleimide (NAM). Samples were incubated for 10 min in loading dye prior to loading to the gel. The electrophoresis was performed at 70 V for 15 min and subsequent 200 V for 1 – 1.5 h with urea-page running buffer (25 mM Tris, 200mM glycine), cooling was applied. The gel was stained in an aqueous solution of 0.1% Coomassie Blue R-250, 50% methanol and 10% acetic acid over-night. The background was destained with an aqueous solution of 10% ethanol and 10% acetic acid.

For the qualitative analysis of the KS catalyzed reaction with urea PAGE, 200 nM KS, 50 µM Mal-ACP and 50 µM C10-ACP or 1.5 µM KS, 180 µM Mal-ACP and 50 µM HB-ACP in MabA buffer were incubated at 25°C. The reaction was stopped by adding equal volume of isopropanol to precipitate the KS. After centrifugation at 20000 x g for 10 min, the supernatant was analyzed as described above.

MabA Assay. All solutions were prepared in 20 µL 384 well microplates (greiner bio-one) and measured using the CLARIOstar Plus platereader (BMG Labtech). The NADPH fluorescence was excited at 348-320 nm and detected at 476-420 nm. All solutions were prepared as 8 x stock in MabA buffer (50 mM sodium phosphate, 10% glycerol, pH 7.0), except of the Mal-X solution, which was prepared as 2 x stock. When adding the Mal-X solution (10 µL) the assay mixture was inverted thoroughly. The substrate titration experiment for wildtype characterization was performed with a knockout protein for a negative control at every condition. The reaction mixture without priming substrate was used as blanks for measurement of low activity (hydroxybutyryl-ACP and crotonyl-ACP) to take priming of decarboxylated malonyl-ACP into account. The fluorescence was measured during a period of 10 min. A final concentration of 5 µM MabA and 50 µM NADPH were used in all measurements. The final concentrations of enzyme and substrates in the assay depended on the experiment and is given in the figure descriptions.

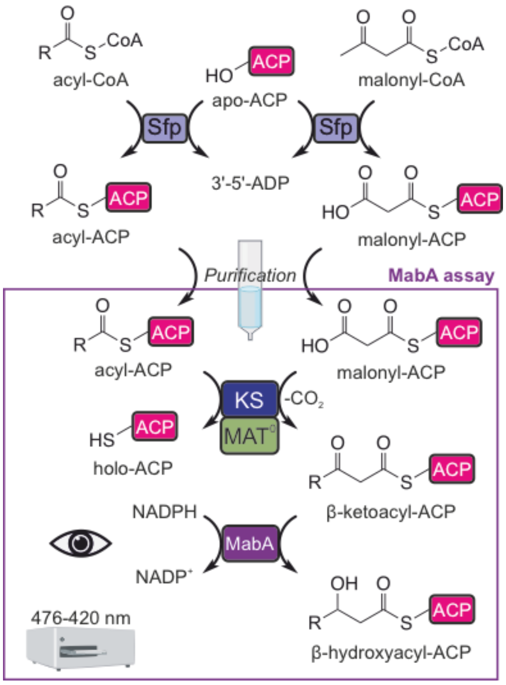

Figure S1: Overview of the MabA assay. The substrates of the MabA assay are generated using the promiscuous Ppant transferase Sfp from *bacillus subtilis*, which transfers the acyl-Ppant or malonyl-Ppant from CoA to apo-ACP. The resulting acyl- and malonyl-ACP was purified from the Sfp and small molecules using Strep affinity chromatography. The purified substrate was applied to the MabA assay together with the KS-MAT^{S581A} (MAT knockout, KS-MAT^{0}), MabA and NADPH. The first step during the assay is the condensation of acyl-ACP and malonyl-ACP, which yields β-ketoacyl-ACP and holo-ACP as a side product. The β-ketoacyl-ACP is subsequently reduced by the reductase MabA. This reduction consumes one molecule NADPH per β-keto-group that is reduced.

Previous assays use absorption spectroscopy,[47] whereas fluorescence was used as readout during this study. The fluorescence of NADPH is excited at 348-320 nm and its emission is monitored by a platereader at 476-420 nm.

Kinetic model. The fit of the sigmoidal data with the Hill equation instead of the Michaelis Menten equation seems logical, as the data shows a sigmoidal shape instead of a hyperbolic curve. To corroborate this interpretation, we fit the data with the following equations for a ping-pong mechanism (Figure S12) and validated the best model with a global F-Test.

Non-cooperative kinetics:

$$v = \frac{v_{max}\,[acyl]\,[malonyl]}{[acyl]\,K_{m,acyl} + [malonyl]\,K_{m,malonyl} + [acyl]\,[malonyl]} \tag{S1}$$

Cooperative kinetics:

$$v = \frac{v_{max}\,[acyl]^h\,[malonyl]^h}{[acyl]^h\,(K'_{acyl})^h + [malonyl]^h\,(K'_{malonyl})^h + [acyl]^h\,[malonyl]^h} \tag{S2}$$

Here, v_{max} is the maximal turnover number, [acyl] is the concentration of the starter substrate/ acyl-ACP, [malonyl] is the concentration of the elongation substrate/ malonyl-ACP, K_m and K′ is the substrate concentration at half-maximal velocity and h is the Hill coefficient. The maximal velocity has a direct relationship with k_{cat}.

$$v_{max} = k_{cat} \cdot [E] \tag{S3}$$

The experimentally determined values [acyl], [malonyl] and v were used to determine the kinetic constants K_m/ K′, k_{cat} and h. Note, that the enzymatic activity (in s^{-1}) used in the fit is the initial velocity (in µM substrate / s) per unit enzyme (in µM) and hence the determined v_{max} = k_{cat}.

To determine which model (non-cooperative or cooperative) fits better to the determined data, the global F-Test (equation S4) was applied (Table S1).

$$F = \frac{(SSR_1 - SSR_2)/\,df_1}{SSR_2\,/\,df_2} \tag{S4}$$

here $df_1 = p_2 - p_1$ and $df_2 = N - p_2$

Here, SSR is the residual sum of squares, df the degrees of freedom, p the number of parameters and N the sample size (4). The subscript 1 and 2 mark values of the non-cooperative model (equation S1) and the cooperative model (equation S2) respectively.

Table S1 Comparison of fits according to equation S1 and S2.

Dataset	***Model***	***SSR***	***F-value***
Acetyl-ACP	Non-Cooperative	0.0029	5.16
	Cooperative	0.0025	
Hexanoyl-ACP	Non-Cooperative	0.0632	73.86
	Cooperative	0.0207	
Decanoyl-ACP	Non-Cooperative	1.8226	121.23
	Cooperative	0.4173	

The F-values off all datasets surpass the F-critical value of the α = 0.05 significance level F_{calc} > F_{tabl} (4.11, α = 0.05), demonstrating that the cooperative interpretation is statistically significant.

Significance of the Hill coefficient h. To estimate the significance of the h coefficients determined by the Hill fit of the wildtype KS and its variants, we applied the approximate ANOVA-Test with the means and variances of determined h-values. The F-value was calculated according to S5-S8 and compared to the F-critical value.

(S5) *grand mean* $$X_G = \frac{\sum(n_i \cdot h_i)}{\sum n_i}$$

(S6) *between group mean square* $$MSB = \frac{\sum n_i \cdot (h_i - X_G)^2}{k-1}$$

(S7) *within group mean square* $$MSW = \frac{\sum(n_i - 1)\cdot {s_i}^2}{\sum n_i - k}$$

(S8) *F value* $$F = \frac{MSB}{MSW}$$

Here, n_i is the number of replicates, h_i the Hill coefficient and s_i is the standard deviation of the dataset i, and k is the number of datasets compared.

The test shows that the means of the three datasets of the wildtype enzyme with respective substrates differ significantly F_{calc} (31.21) > F_{tabl} (5.14, α = 0.05), here the error determined from the fit is treated as standard deviation s. The differences between the mutants, however, do not proof to be significant F_{calc}(1.87) < F_{tabl}(4.07, α = 0.05).

To estimate if the determined h-values of the variants are significantly higher than $\mu_0 = 2$ (expected maximal value of a dimeric protein with traditional cooperative mechanism), we applied the one-sample one-tailed t-test (equation S9). We tested the hypothesis as follows: H_0: $X = \mu_0$ and H1: $X > \mu_0$, here X is the mean of the h-value determined in the study of the mutants.

(S9) $$t = \frac{X - \mu_0}{\frac{s}{\sqrt{n}}} \quad \text{and} \quad df = n - 1$$

Here, s is the standard-deviation and n the sample size. The determined t-value for the wildtype in respective study is t_{calc}(2) > t_{tabl}(1.64, α = 0.1) at df = 3. The t-value for the R137K is calculated as t_{calc}(2.97) > t_{tabl}(2.92, α = 0.05) and for R137 as t_{calc}(1.93) > t_{tabl}(1.89, α = 0.1) at df = 2. Hence, the h-values for the wildtype are higher than $\mu_0 = 2$ at low significance level α = 0.1.

Calculation of transition state energies.
The transition state energy and the turnover number of an enzyme catalyzed reaction exhibits following relationship according to the transition state theory:

(S10) $$\Delta G^{\ddagger} = -RT \ln\left(\frac{k_{cat} \cdot h}{k_B \cdot T}\right)$$

Here, $\Delta G^{\ddagger}$ is the Gibbs free energy of the transition state, k_{cat} the rate constant (turnover number), R gas constant, h the Planck constant, k_B the Boltzmann constant and T the temperature (298.15 K). According to this relationship the transition state energies of the KS can be compared for different substrates:

$\Delta G^{\ddagger}$ (Acetyl-ACP) = 76 kJ/mol
$\Delta G^{\ddagger}$ (Hexanoyl-ACP) = 74 kJ/mol
$\Delta G^{\ddagger}$ (Decanoyl-ACP) = 70 kJ/mol
$\Delta G^{\ddagger}$ (Crotonyl-ACP)* = 85 kJ/mol
*The calculation is based on the apparent turnover number of crotonyl-ACP k_{cat} = 0.0081 s^{-1}.

Hexanyol-SNAC synthesis. This synthesis was adopted from Peter et al. and Valenzano et al.[67,68] 0.6 mL triethylamine (4.3 mmol, 2 eq.) was added to the hexanoic acid (4.3 mmol, 2 eq.) in 20 mL tetrahydrofurane (THF) (dry) at 0°C. 0.4 mL ethyl chloroformate (4.3 mmol, 2 eq.) was added and the suspension was stirred excessively under argon for 45 min at 0°C. The suspension was filled into 50 mL tubes and centrifuged for 10 min at 4°C and 3220 x g to remove insoluble salts. About 20 mL of the supernatant was added to 230 mL SNAC (2.2 mmol, 1 eq.) in 10 mL 0.1 M NaHCO3 (pH 8) and stirred for 1 h at room temperature. The product was extracted 3-4 times from the resulting solution with 30 mL diethyl ether each time. The organic phase was washed 3 times with 10 mL 0.1 M Na_2CO_3 (pH 9) each time and 5 mL sodium chloride solution (sat.), dried with magnesium sulfate and filtered. The solvent was removed using a rotary evaporator. The raw product was further purified by column chromatography using EE:H 1:1 to 3:1. Hexanoyl-SNAC was obtained as colorless solid (89 mg, 20%, R^f (EE:H 4:1) = 0.26, m/z [M+Na] = 240.04). 250 MHz, $CDCl_3$ δ[ppm]: 5.68 (s, NH), 3.44 (td, $^3J_{HH}$=6.1 Hz, $^3J_{HH}$=6.1 Hz, 2H, CH_2-2), 3.02 (t, $^3J_{HH}$=6.4 Hz, 2H, CH_2-3), 2.57 (t, $^3J_{HH}$=7.5 Hz, 2H, CH_2-4), 1.97 (s, 3H, CH_3-1), 1.66 (quint, $^3J_{HH}$=7.3 Hz, 2H, CH_2-5), 1.36 - 1.25 (m, 4H, CH_2-6 - CH_2-7), 0.90 (t, $^3J_{HH}$=6.8 Hz, 3H, CH_3-8). A contaminant between 1.6 and 1.8 ppm could not be identified. The interpretation of measured NMR spectra were supported by the literature.[69,70]

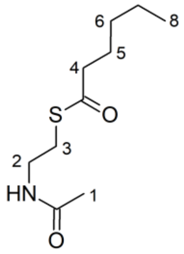

Thermal fluorescence assay. The stability of mutants was assayed in five different buffers that are used throughout the purification and evaluation procedure. A solution of around 2 µg protein in His-wash buffer, His-elution buffer, Strep-wash buffer, strep-elution buffer and MabA buffer was prepared on ice in 96-well plates (Multiplate® PCR Plates™, Bio-Rad) and Sypro™ Orange (Invitrogen) was added according to manufacturer's guideline. The well plate was sealed with optical tape (iCycler iQ®, Bio-Rad) and centrifuged shortly at 3000 xg. The measurements were performed in a real-time thermocycler (CFX Connect™, Bio-Rad) at excitation/emission wavelengths of 450-490/650-580. A stepwise temperature increase of 0.5°C/30s was performed between 5°C and 95°C.

System Preparation. The crystallographic x-ray structure of murine FAS KS-MAT domain (PDB ID: 6ROP, 2.70 Å resolution)[24] was used to prepare the KS-substrate models used in this study. The asymmetric unit comprises four copies of the KS-MAT di-domain arranged in two functional dimers MAT-KS:MAT-KS (chains A-B:C-D).
Two adjacent KS domains (residues 1-409 plus 825-852) from chains A and B were extracted and used to build the studied model. There were no water molecules present in the structure. Furthermore, this structure had an octanoyl moiety covalently bounded to the KS domain that was deleted before modeling the substrate in the KS active site.

The acetyl moiety carbonyl oxygen was oriented to the backbone amides of Cys161 and Phe395, which are expected to act as an oxyanion hole during the KS-catalyzed reaction.[22,24] The catalytic cysteine (Cys161) was bonded to the octanoyl moiety in the x-ray structure. This residue was oriented to His331 to approximate the KS active site to a catalytically competent conformation in the prepared system.

The H++ webserver[71] was used to predict the protonation states of the titratable residues. According to H++, Cys161 should exist in neutral form (-SH). The protonation of the histidines was carefully manually checked, and the predictions from H++ were accepted except for His293, which was protonated in its δ nitrogen.

The coordinates from PaM10 were saved and transferred to the 6ROP active site. The GaussView software was used to convert the PaM10 into a Ppant group and to add an acetyl moiety to its terminal thiol group, generating the acetyl-Ppant substrate. The coordinates of the introduced acetyl moiety were designed to maximize the hydrogen bonds between its carbonyl oxygen and the backbone amides of Cys161 and Phe395, which are expected to form an oxyanion hole throughout the KS-catalyzed reaction. It is worth noting that substrates containing butyryl, C6acyl, C10acyl, crotonyl, and hydroxybutyryl were also created using the same methodology to obtain Ppant substrates.

The enzyme residues were parameterized using the AMBER ff14SB force field. The substrates were parameterized using the following protocol: First, they were divided into two distinct molecules, Ppant and the substrate moieties, which were parameterized as independent units. Hydrogen atoms were added to the carbonyl carbon of the substrate molecules and to the terminal sulfur atoms of the Ppant group to complete their valence shells. The Antechamber module of the Amber 18 package was used to parameterize the independent units with GAFF2. The charges were derived from a RESP fitting of the electrostatic potential determined at HF/6-31G(d) level of theory. The charge of the added hydrogen atoms was kept equal to zero and constant throughout the calculation of the electrostatic parameters for each molecule. The Xleap module of the Amber 18 package was used to delete the added hydrogen atom and to create a covalent bond between the substrate moiety and Ppant molecules. Xleap was also used to add Na^+ counterions and solvate the system with an octahedral box of TIP3P water molecules within a radius of 12 Å from the surface of the protein.

MD Simulations for substrate binding. The energy of the prepared systems was minimized to alleviate steric clashes or unfavorable tensions that may be present in the modeled systems. Gromacs 2021 was employed, and the minimization was performed in two steps. First, the water molecules, hydrogens, and counter-ions were minimized using the steepest descent algorithm. Periodic boundary conditions were imposed to account for long-range interactions. A radius of 10 Å was defined as the cut-off distance for short-range electrostatic and Lennard-Jones interactions. The entire system was minimized in the following stage using the same conditions.

The system was then equilibrated, starting with a 100 ps simulation with an NVT ensemble using the modified Berendsen thermostat and a reference temperature of 300 K. Then, a 100 ps equilibration MD was run with the NPT ensemble, in which the density of the system was equilibrated at 300 K and 1 bar using the modified Berendsen thermostat and the Berendsen barostat. During these stages, the protein and the substrates were constrained with positional restraints of 1000 kJ mol^{-1} nm^{-2} and 2000 kJ mol^{-1} nm^{-2}, respectively.

The equilibration phase was followed by a 50 ns simulation run with the NPT ensemble in which the hydrogen atom H17 and the oxygen atom O7 from Ppant and three Thr residues (Thr262, Thr295, and Thr297) from the protein active site were restrained with a force of 1000 kJ mol^{-1} nm^{-2}, to guarantee the initial orientation of the substrate through the establishment of hydrogen bonds between the threonines and the atoms from Ppant. Finally, an unrestrained production phase of 50 ns using the NPT ensemble was conducted.

Modelling of the *Rattus norvegicus* FAS. The *Rattus norvegicus* FAS (rFAS) that served as the foundational template was sourced from AlphaFold (AF) with the corresponding code AF-P12785-F1. As the AlphaFold entry revealed the specific residues responsible for forming the

linker between the Ketoacyl Reductase (KR) and Acyl Carrier Protein (ACP) domains, homology modelling was implemented to construct the linker.
First, the thioesterase (TE) and ACP domains were deliberately excluded from the AF-P12785-F1 template. The ACP domain was subsequently incorporated back into the structure using an AlphaFold model of the KS:ACP complex, obtained from an AlphaFold Colab calculation.[72]
Except for the absent TE domain, the target FAS sequence was submitted to the SWISS-Model software for model construction.[73]
The geometry of the modelled FAS was then optimized with the Sander module of the Amber software package through 1000 steepest descent steps and 1500 conjugate gradient steps.[74]

Parameterization of the *Rattus norvegicus* FAS. The final system was built on the tleap program using an AMBER ff14SB force field and TIP3P water molecules. A cuboid box of water molecules with faces at a minimum distance of 10 Å from the protein was created to solvate the protein. Furthermore, 61 Na+ counterions were added to neutralize the system's total charge. Given the large size of the modelled FAS protein (4404 residues) and the simulation box necessary to simulate it, the total system was very large, consisting of 924340 atoms, limiting the length of the subsequent MD simulations.
The protonation state of ionizable residues was estimated using the web server PDB2PQR to predict their local pKa values. The AMBER parameter files were converted to GROMACS parameters using a Python script and the amber.python command, as the molecular dynamics simulation was conducted using the GROMACS software.

MS Simulations for ACP binding. A molecular dynamics simulation of 200 ns was conducted using the GROMACS software. At first, the system underwent a two-stage minimization process, initially addressing the water molecules and then minimizing the entire system. Subsequentially, the complex was equilibrated for 20 ns in the canonical (NVT) ensemble with restraints applied to all the protein atoms except the ACP:KR linker. In the next step, an equilibration was performed with positional restraints imposed on the ACP:KS interfacial residues (45-49, 198-205, 297-298 of KS and 47-74 of ACP) lasting 200 ns. In both equilibration phases, conducted within the NVT ensemble, a V-rescale (modified Berendsen) thermostat was utilized. The subsequent and final molecular dynamics run was done at the isothermal-isobaric (NPT) ensemble, using the Berendsen barostat without positional restraints, for 200 ns. Therefore, a total simulation time of 420 ns was run.
The reference temperature for all MD simulations was set to 310.15 K.
In one of the replicas, the NPT run was prolonged up to 1 µs employing the Parrinello-Rahman thermostat.
Therefore, a total of 1.84 µs of MD simulation was run. RMSD values were then calculated using analysis tools within the GROMACS software.

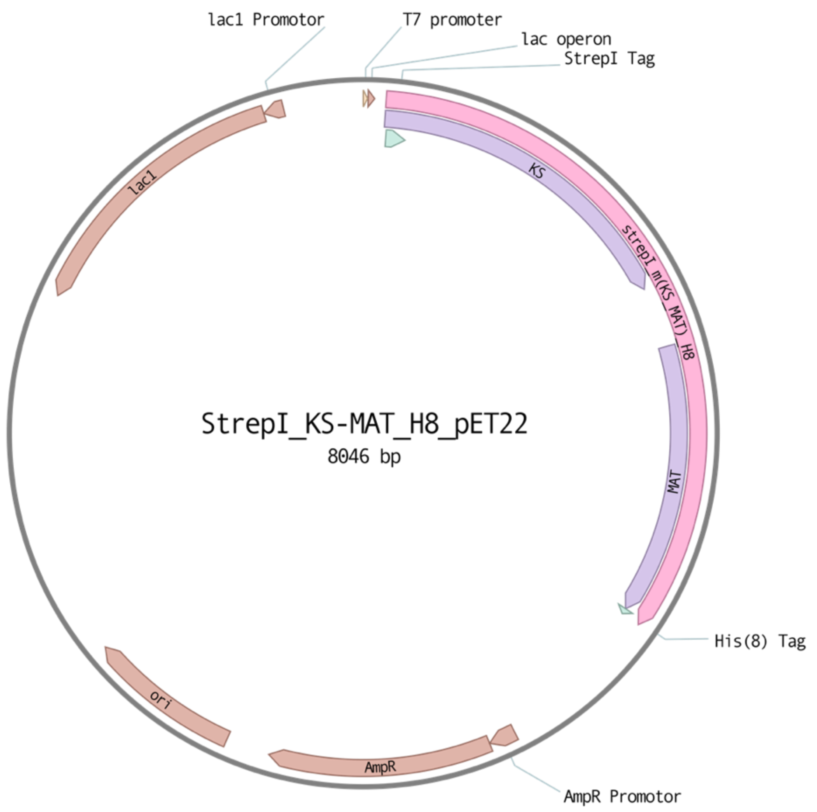

Figure S2: Plasmid map of KS-MAT. Vector sites are presented in brown, including lac operon (lac1), origin of replication (ori) and ampicillin resistance (AmpR). The gene encoding for the KS-MAT is shown in pink with the KS and MAT structural part shown in violet respectively. The N-terminal StrepI-Tag and C-terminal 8xHis-Tag are shown in green.

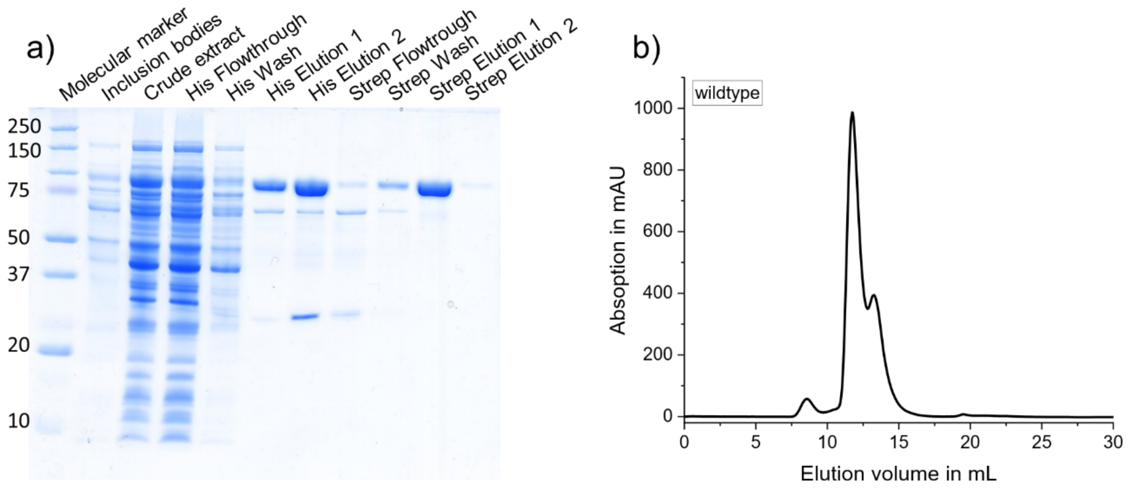

Figure S3: Purification and quality control of KS-MAT^{S581A}. a) SDS PAGE of of the purification procedure using tandem affinity chromatography. The target protein has a size of around 97 kDa. b) Size exclusion chromatogram shows aggregation, that elute at around 8 mL, dimer at around 11 mL and monomeric KS-MAT at around 13 mL.

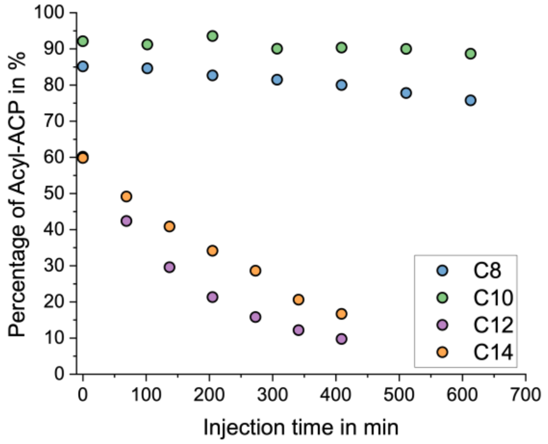

Figure S4: Hydrolysis of Acyl-ACPs. Freshly prepared Acyl-ACPs were analyzed after different time with HPLC. The peak integrals of octanoyl-ACP, decanoyl-ACP, lauryl-ACP and myristyl-ACP are shown in dependance of the hydrolysis time. The samples were maintained at tempered at 4°C during this measurement series.

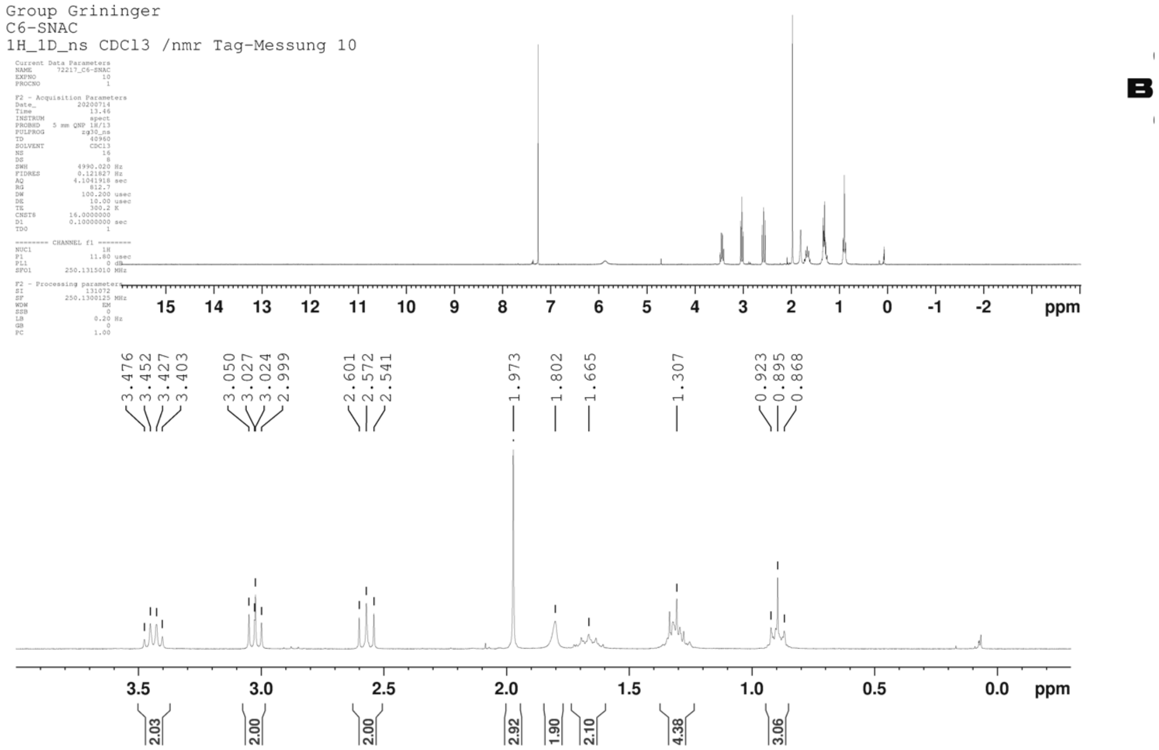

Figure S5: NMR spectrum of hexanoyl-SNAC. Top: Spectrum over a wide range. Bottom: Zoom to the range of product signals. (250 MHz, $CDCl_3$)

a) acetyl-CoA

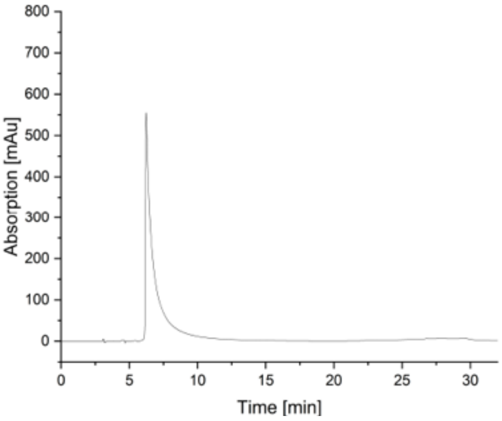

b) hexanoyl-CoA

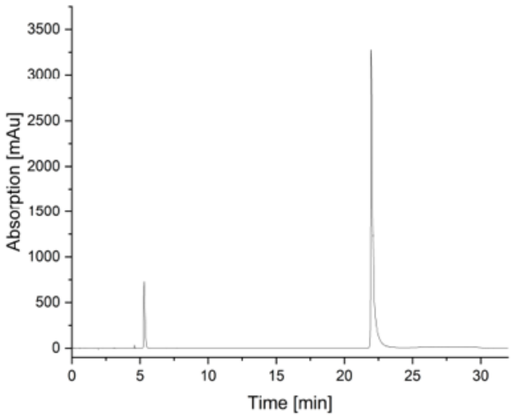

d) octanoyl-CoA

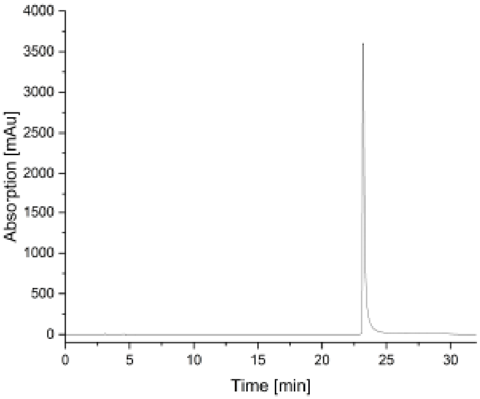

c) decanoyl-CoA

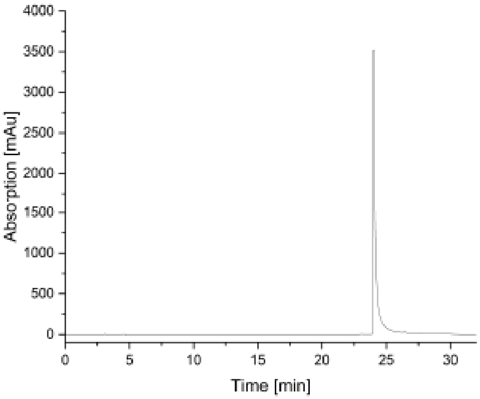

f) lauroyl-CoA

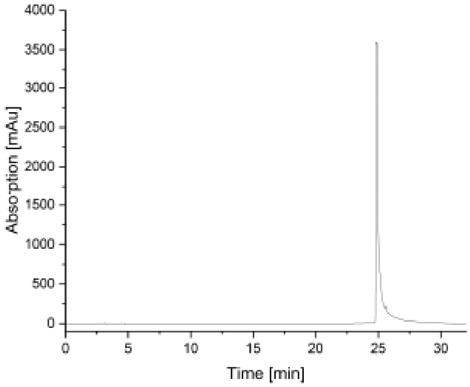

e) myristoyl-CoA

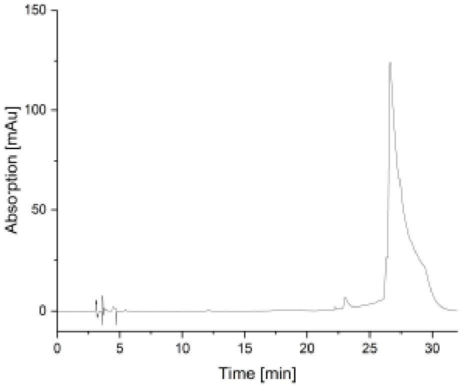

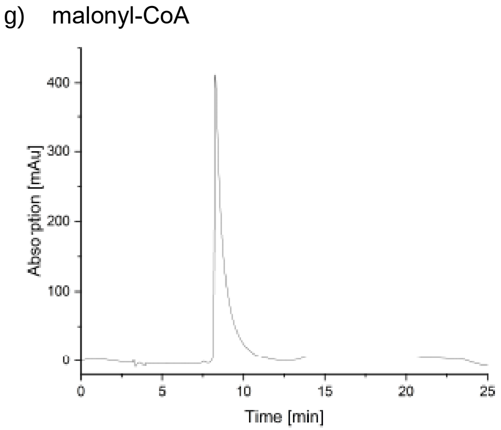

Figure S6: Representative HPLC chromatograms for acyl-CoA esters.

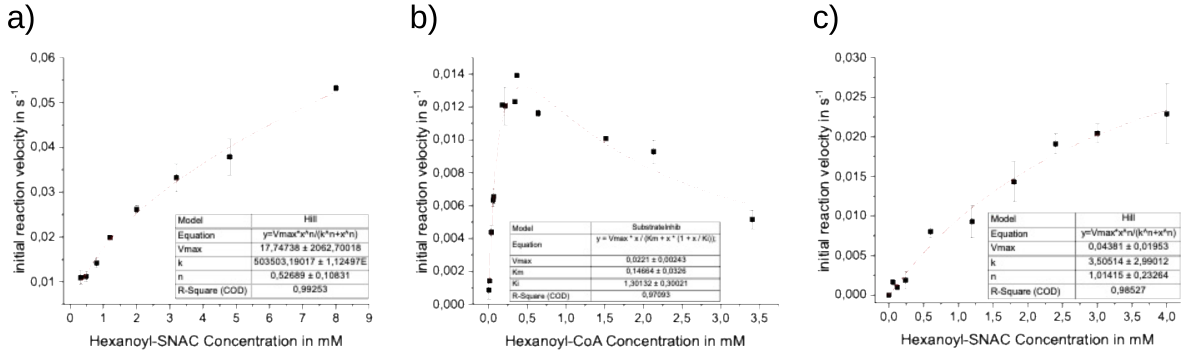

Figure S7: Carrier Analysis a) The wildtype KS was assayed at 1.7 µM protein and 250 µM malonyl-CoA. The data was fit to the Hill equation to take a potential cooperativity into account. b) The wildtype KS was assayed at 1.9 µM protein and 470 µM malonyl-CoA. The data was fit to the substrate inhibition equation, because the activity decreased significantly with increasing substrate concentration. c) The wildtype KS was assayed at 1.4 µM protein and 47 µM malonyl-ACP. The data was fitted to the Hill equation to take potential cooperativity into account. All data fits were performed using OriginLab®.

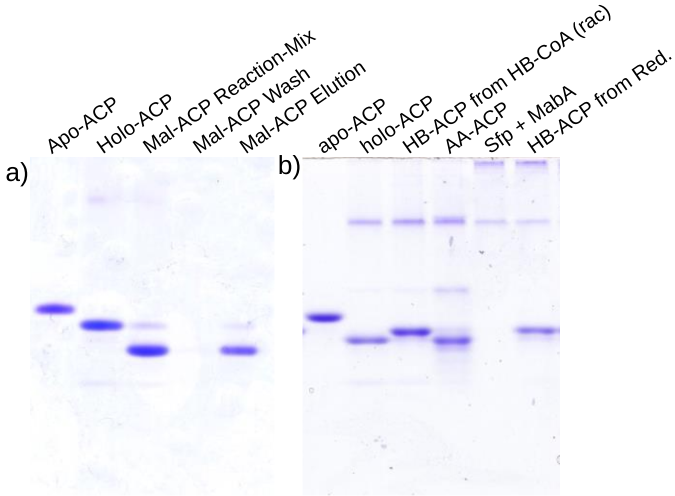

Figure S8: Urea polyacrylamide gels of X-ACPs. a) NEM was included in the loading dye. Apo-ACP and holo-ACP are shown as reference. The reaction-mix of Mal-ACP generation includes apo-ACP, Sfp and Mal-CoA. The wash fraction and the elution fraction according to the purification protocol are shown. Mal-ACP was acquired in

90% purity with small amounts of holo-ACP from thioester hydrolysis. b) NAM was included in the loading dye. Apo-ACP and holo-ACP are shown as reference. HB-ACP was generated in a Sfp catalyzed reaction from apo-ACP and HB-CoA (rac). AA (acetoacetyl)-ACP was generated from apo-ACP and AA-CoA. Finally, (R)-HB-ACP was generated by phosphopantetheinylation from apo-ACP and AA-CoA with subsequent reduction of MabA with NADPH. (R)-HB-ACP was acquired in around 90% purity.

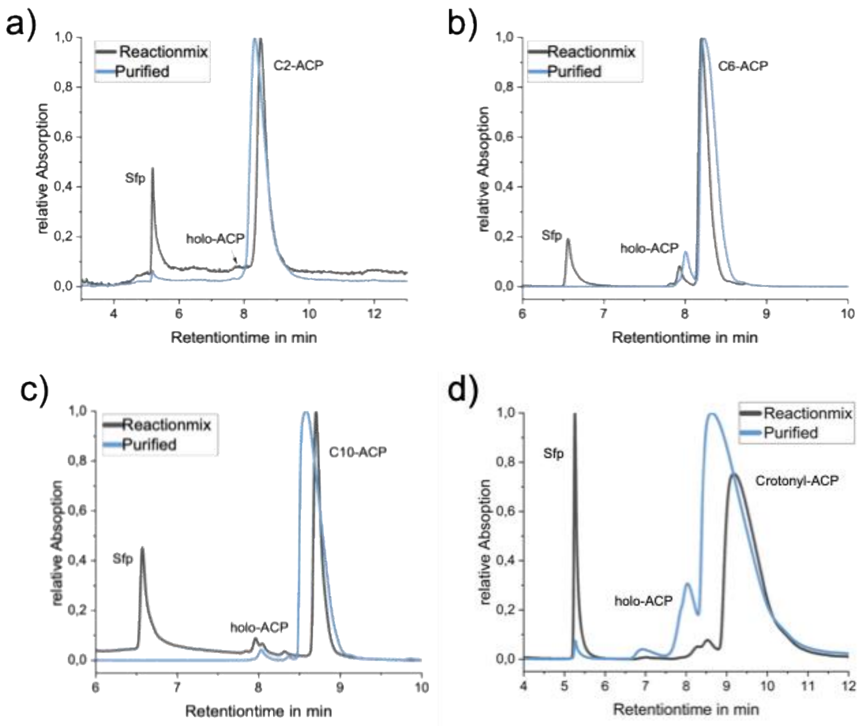

Figure S9: HPLC chromatograms of Acyl-ACPs. a) Acetyl-ACP b) Hexanoyl-ACP c) Decanoyl-ACP and d) Crotonyl-ACP. Black: Reaction mix of acyl-ACP generation including Sfp, apo-ACP and respective acyl-CoA. Blue: Purified acyl-ACP after strep-tactin-column purification and rebuffering in ACP buffer. Peaks are normalized.

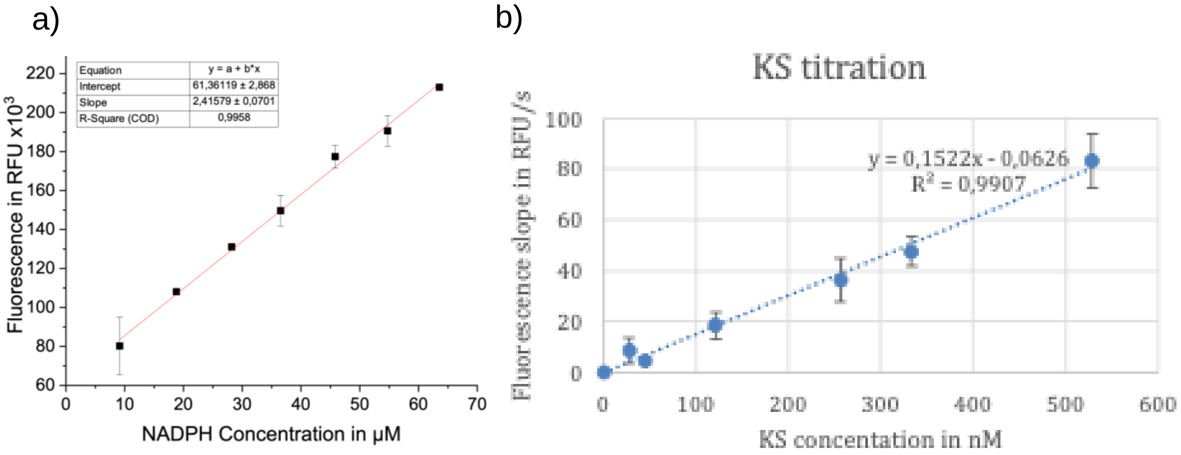

Figure S10: Representative calibration and KS titration. a) Dependence of NADPH fluorescence on concentration is measured with the same settings and under the same conditions as the final KS activity measurements. b) Different concentrations of KS were applied to the MabA assay to ensure, that the KS mediated reaction is not rate-limiting in the assay setup. As final concentrations 5µM MabA, 120µM Mal-ACP and 120µM C2-ACP were used. Datapoints represent the mean and error bars the standard deviation of technical replicates.

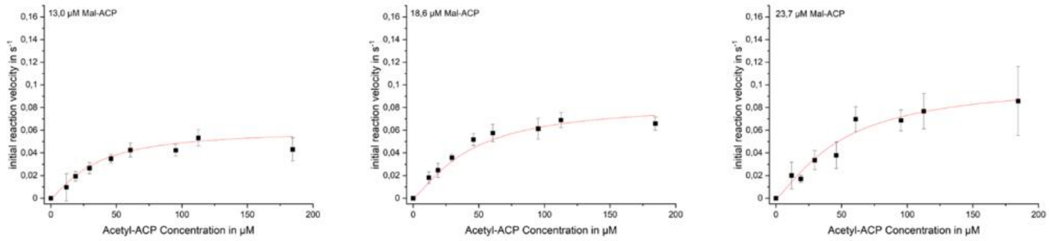

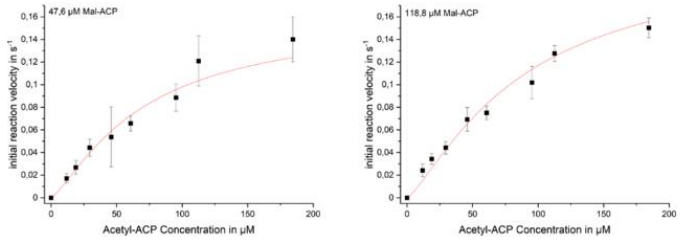

Constant	Acetyl-ACP	Malonyl-ACP
K_m/ µM	100 ± 26	33 ± 7
K_{cat}/ s^{-1}	0,26 ± 0,05	0,26 ± 0,05
K_{cat}/K_m	0,0026 ± 0,0008	0,007 ± 0,002
h	1,28 ± 0,11	1,28 ± 0,11

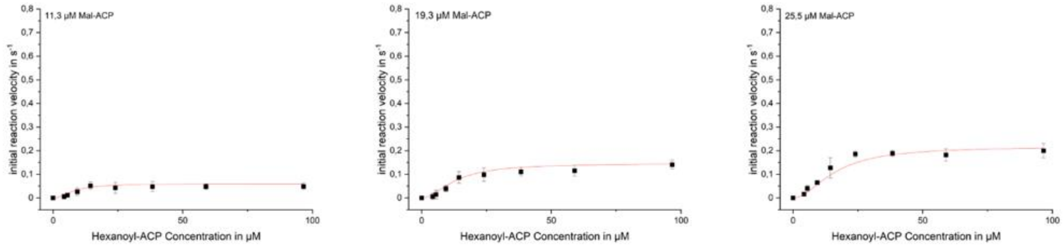

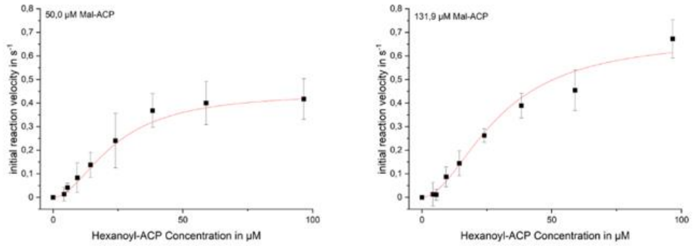

Constant	Hexanoyl-ACP	Malonyl-ACP
K_m/ µM	31 ± 3	41 ± 3
K_{cat}/ s^{-1}	0,75 ± 0,05	0,75 ± 0,05
K_{cat}/K_m	0,024 ± 0,003	0,018 ± 0,002
h	1,90 ± 0,11	1,90 ± 0,11

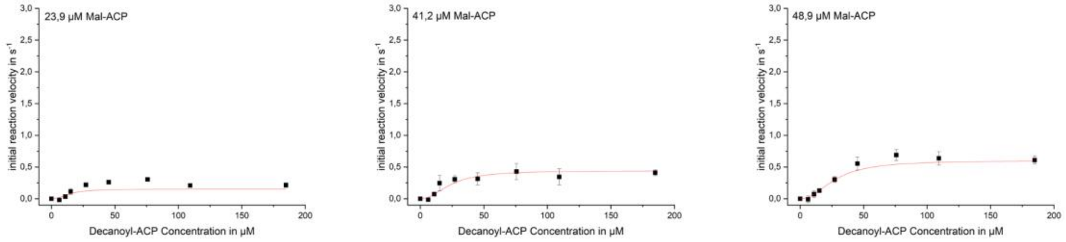

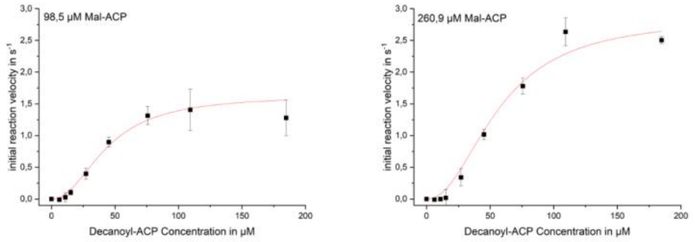

Constant	Decanoyl-ACP	Malonyl-ACP
K_m/ µM	60 ± 5	97 ± 8
K_{cat}/ s^{-1}	3,2 ± 0,2	3,2 ± 0,2
K_{cat}/K_m	0,053 ± 0,006	0,033 ± 0,003
h	2,1 ± 0,1	2,1 ± 0,1

Figure S11: Kinetic characterization of the wildtype KS using the MabA assay. The initial reaction velocity is plotted against eight different concentrations of acyl-ACP at five malonyl-ACP concentrations. The KS concentration for all measurements was 300 nM. Individual measurement series were performed for each of three carbon chain lengths. Datapoints represent the mean and error bars the standard deviation of three biological replicates. The obtained titration curves were globally fitted for each chain length using the Hill equation without any constraints. The kinetic constants are provided in the respective tables. The enzymatic efficiencies k_{cat}/K' are given in $s^{-1}µM^{-1}$. a) acetyl-ACP b) hexanoyl-ACP c) decanoyl-ACP.

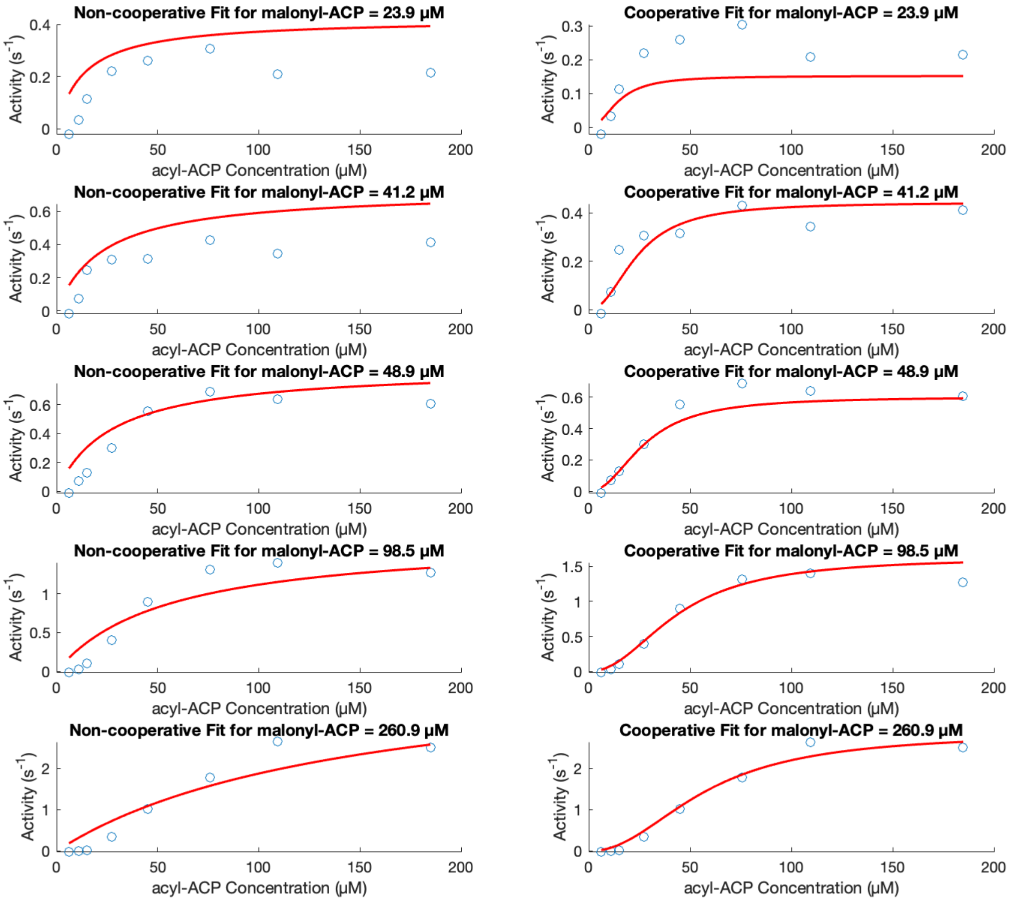

Figure S12: Fit functions in comparison for the decanoyl-ACP titration. Left: data fit assuming non-cooperative kinetics according to equation S1 Right: data fit assuming cooperative kinetics according to equation S2. This fit was performed with MATLAB and does not include datapoints at 0/0, weights or any constraints.

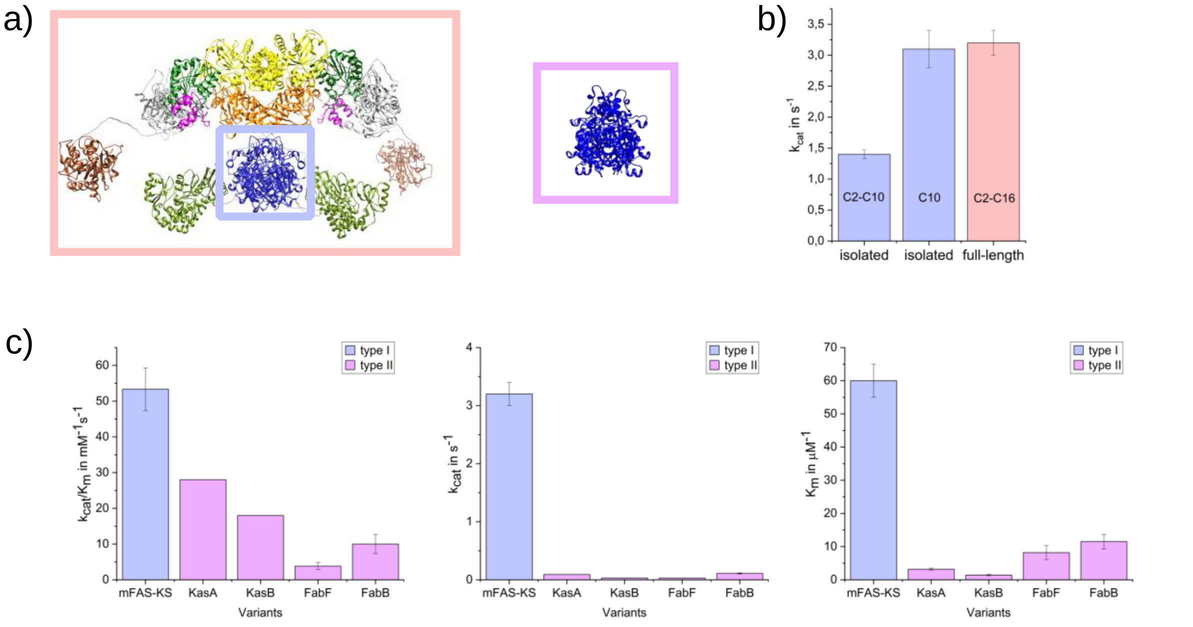

Figure S13: Enzymatic constant comparison a) Schematic depiction of mFAS (orange), mFAS-KS (blue) and type II KS (pink) (PDB: 1G5X)[23, 75] b) comparison of k_{cat} of isolated mFAS-KS (blue) and full-length FAS (orange). c)-e) Enzymatic constants of isolated mFAS-KS (blue) and type II bacterial KS (pink). Apparent enzymatic constants of KasA and KasB refer to C16-ACP, FabF and FabB data were obtained with C14-ACP. [35,45]

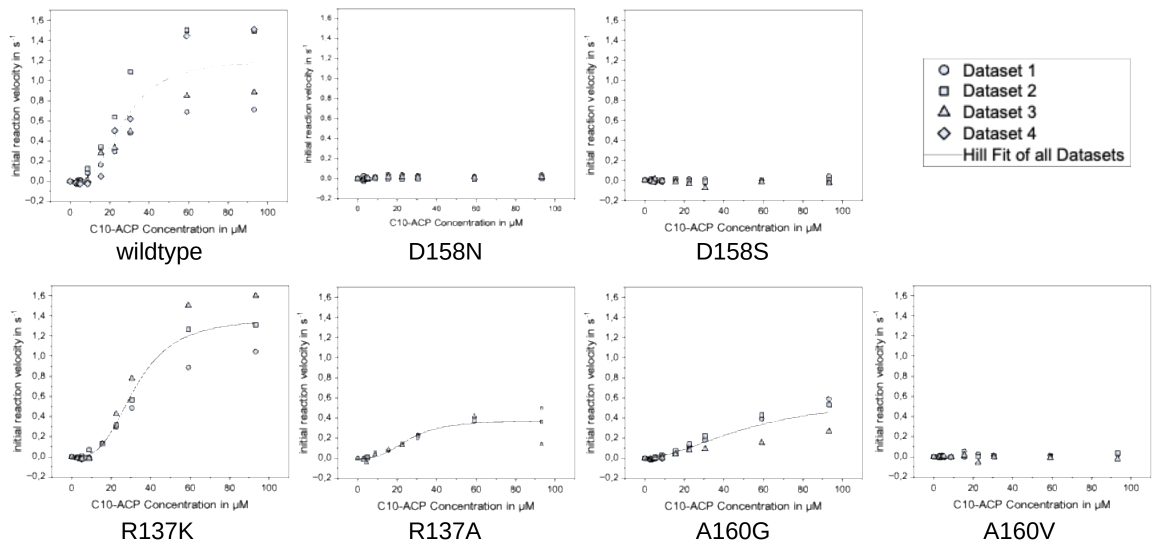

Figure S14: Titration of C10-ACP in titration measurements with mutants. The measurements were performed with 300 nM KS at 50 µM Mal-ACP. The data was fitted with the Hill equation using OriginLab®. The results of the fit can be found in table S2.

Table S2. The values derived from the titration experiment of mutants, that attempted to delete the hydrogen bond network and thus intersubunit communication.

**Protein**	**h**	**$v_{max}^{app.}$/ s^{-1}**	**$K_m^{app.}$/ µM**
wildtype	2.9 ± 0.9	1.21 ± 0.12	27 ± 3
D158N	not active		
D158S	not active		
R137K	3.2 ± 0.7	1.38 ± 0.10	32 ± 3
R137A	3.0 ± 0.9	0.38 ± 0.04	26 ± 3
A160G	1.8 ± 0.6	0.6 ± 0.3	60 ± 31
A160V	not active		

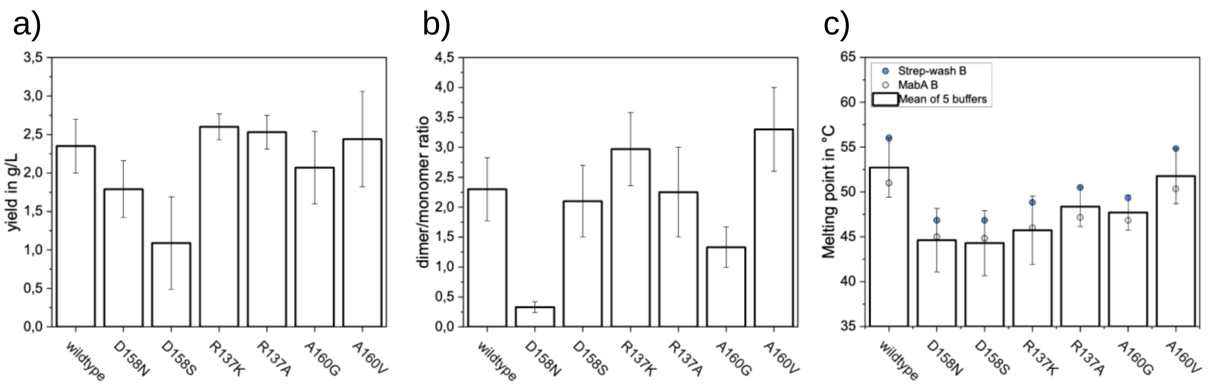

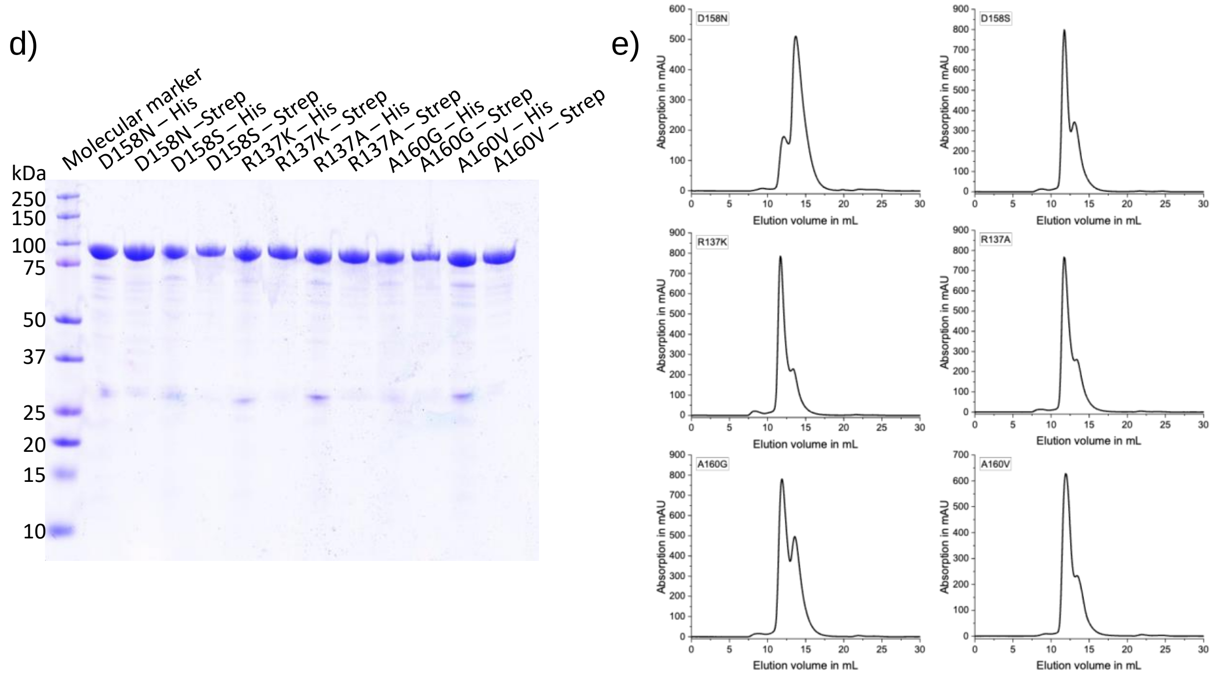

Figure S15: Analysis of the protein quality of mutants that were used to investigate the enzyme allostery. a) The average of the protein yields of three expressions in mg protein per liter of main culture. b) The average of dimer/monomer ratio obtained for three biological replicates from the size exclusion chromatogram. c) Thermal fluorescence assay was performed for all mutants in five buffers, which are used in the workflow of protein preparation and kinetic evaluation. The minimum of the melting point derivative is given as melting point. The storage buffer (Strep-wash B) and assay buffer (MabA B) are shown as separate data points. All datapoints represent biological triplicates. d) Exemplary SDS PAGE analysis of the elution fractions of of Ni-NTA column and Strep-Tactin column of all mutants. e) Representative size-exclusion chromatograms of all mutants.

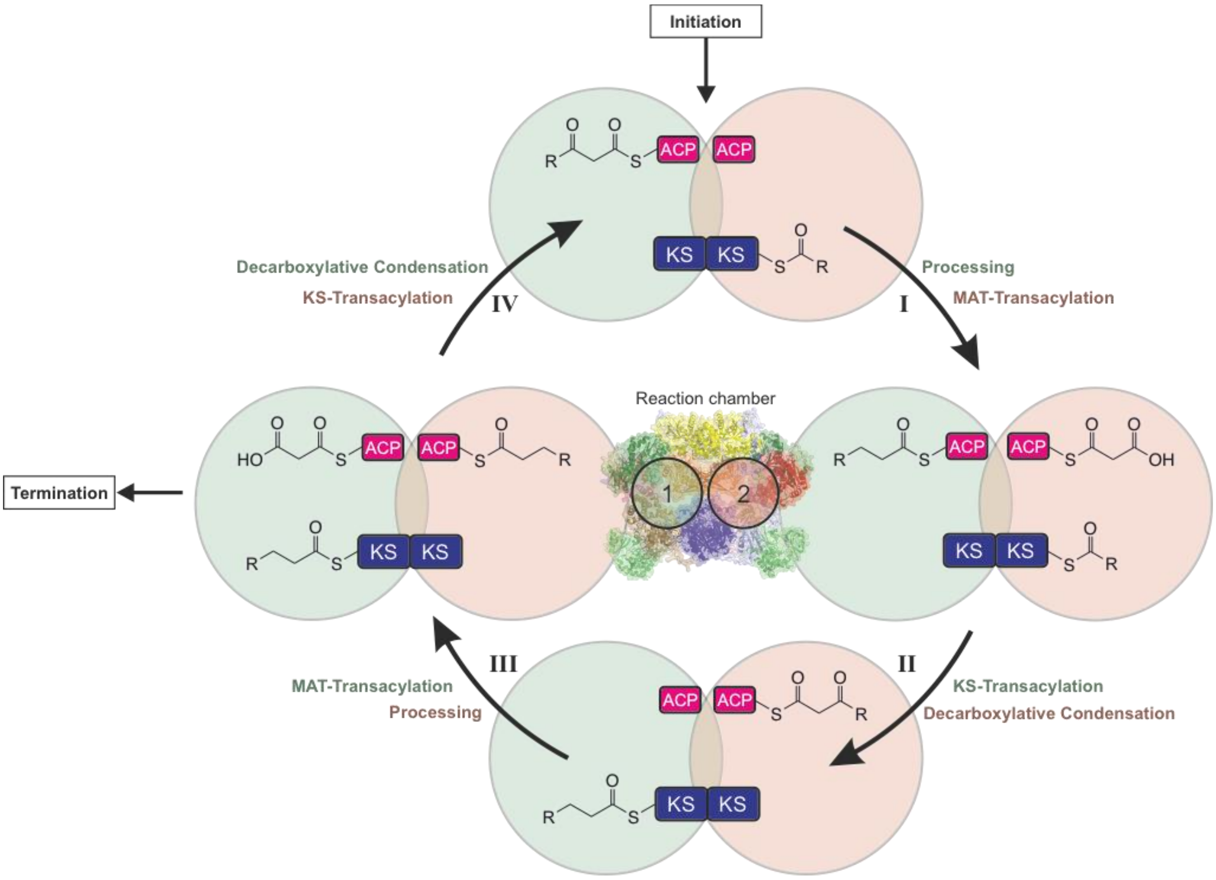

Figure S16: Proposal of an asynchronous fatty acid cycle model. The illustration shows snapshots of two ACP and KS active site in a speculative fatty acid cycle. The two reaction chambers of the FAS are colored in

green and red respectively. The carrier analysis of the starter substrate and the extender substrate revealed that both substrates are involved in the cooperative mechanism of the FAS indicating that the PING and PONG step of the KS catalysis occur in parallel. The alternation of parallel PING-PONG-KS catalysis with parallel Transacylation-Processing catalysis (involving MAT and KR-DH-ER respectively) would lead to an efficient fatty acid cycle. The steps II and IV would be promoted by the cooperativity of the KS.

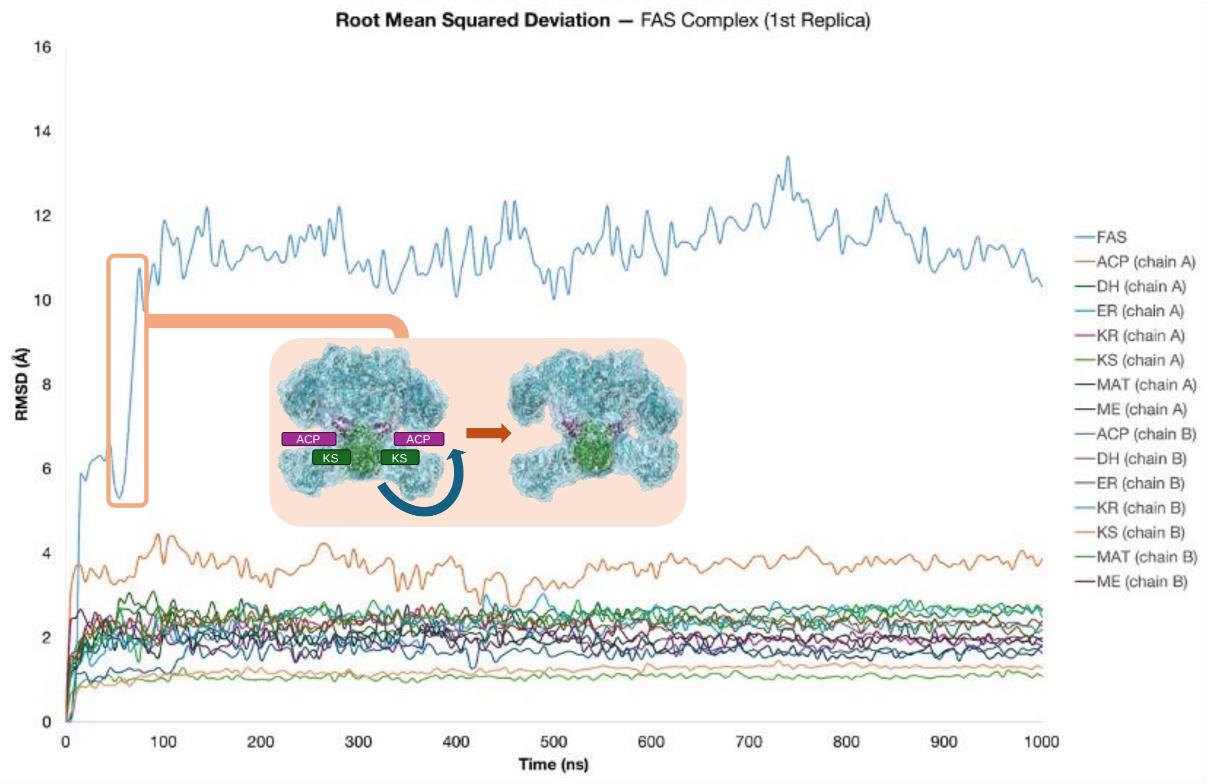

Figure S17: The Root Mean Square Deviation (RMSD) of the FAS complex and its individual domains from both polypeptide chains in the first replica. Running averages of the RMSD values were calculated to facilitate the visual analysis of the complex and its domains' stability. The different simulation steps are depicted, emphasizing the conformational shifts observed during the equilibration simulations. The graphic shows that the rFAS adopts an asymmetrical conformation, simultaneously binding both ACP domains to KS. The asymmetric conformation is reached through rigid-body domain motions, as confirmed by the low RMSD of the individual domains. A visual representation of the conformational shift is provided for clarity, with the ACP and KS domains highlighted in magenta and green, respectively.

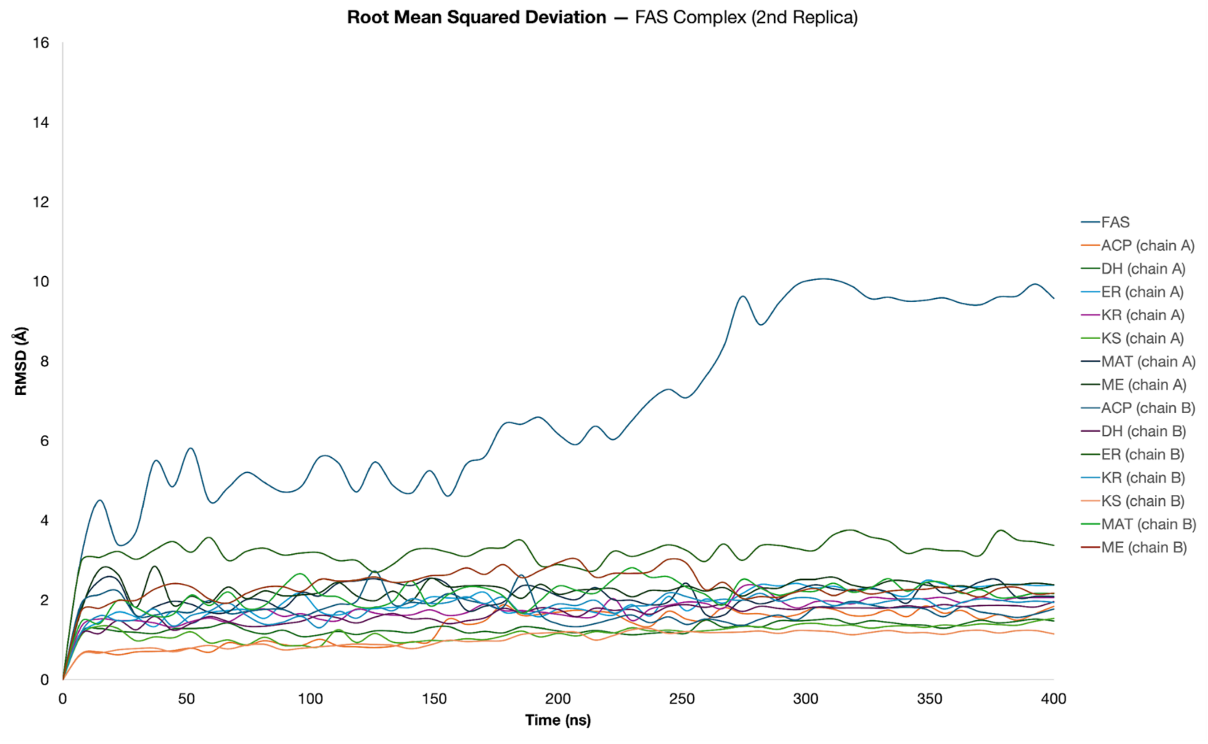

Figure S18: The Root Mean Square Deviation (RMSD) of the FAS complex and its domains from both polypeptide chains in the second replica. Running averages of the RMSD values were calculated to facilitate the visual analysis of the complex's and domains' stability.

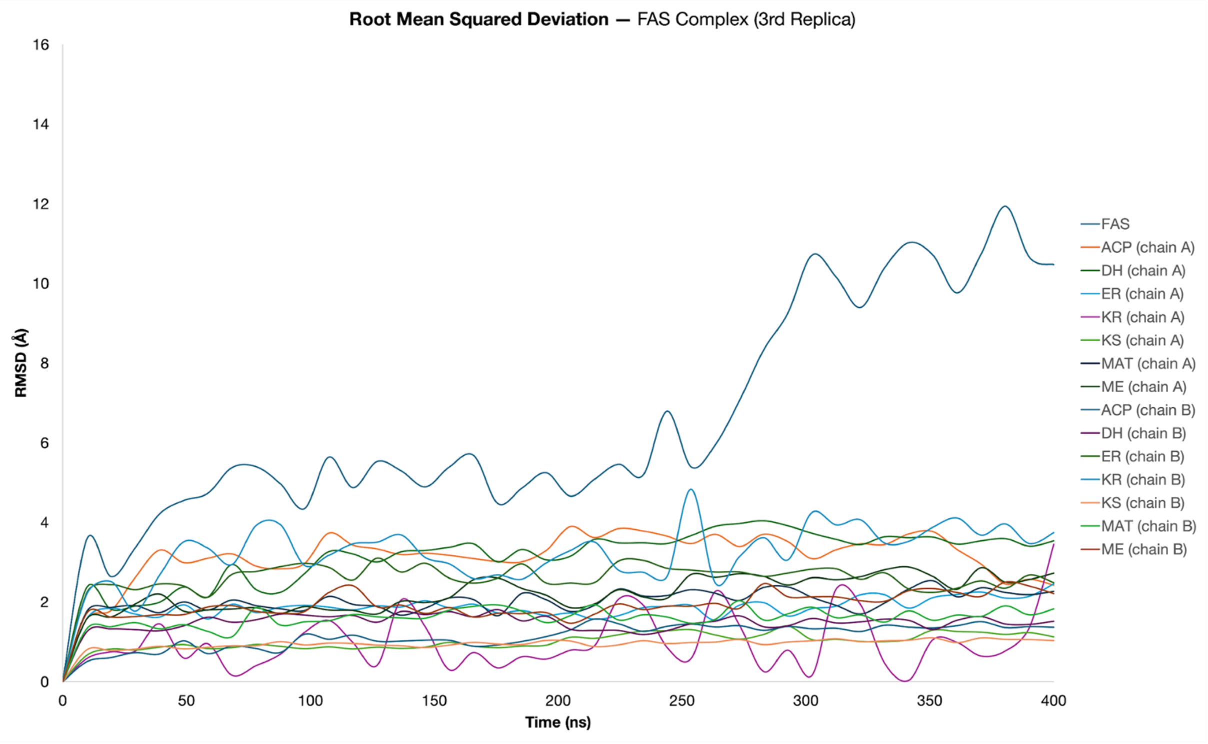

Figure S19: The Root Mean Square Deviation (RMSD) of the FAS complex and its domains from both polypeptide chains in the third replica. Running averages of the RMSD values were calculated to facilitate the visual analysis of the complex and its domain stability. In this third replica, as ACP dissociates from KS, the RMSD values exhibit reduced stability, indicating increased flexibility of ACP within the overall system. The dissociation observed after the conformational shift in the third replica is within expectation, as ACP is anticipated to dissociate easily to facilitate the continuation of the FAS cycle.

Table S3. The linker length between the ACP and KR domains for both rFAS chains. The distance between the residue's alpha-carbon at the start and end of the linker was computed. Mean values and standard deviations are provided for each chain. The differences in chain length between the replicas illustrates the conformational flexibility of the rFAS multienzyme and the diversity of structural solutions for binding both ACP domains to KS simultaneously.

Replica	Chain A (Å)	Chain B (Å)
1st Replica	43.1 ± 0.9	45.5 ± 0.8
2nd Replica	38.3 ± 0.8	33.2 ± 1.0
3rd Replica	34.5 ± 0.6	30.0 ± 0.6

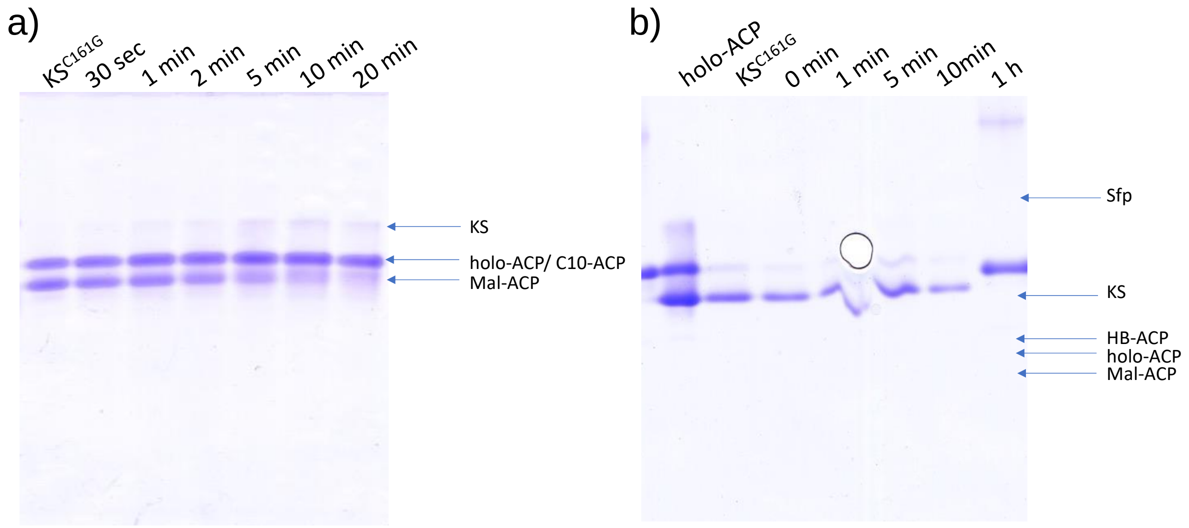

Figure S20: Urea PAGE analysis of KS catalyzed condensation reaction. a) The gel shows the time course of the reaction of decanoyl-ACP with malonyl-ACP in presence of KS. Negative control includes KS^{C161G} as a functional knockout. Note that decanoyl and holo-ACP cannot be separated under the chosen conditions. The reaction progress is monitored by the amount of malonyl-ACP. b) The gel shows the time course of the reaction of (R)-hydroxybutyryl-ACP with malonyl-ACP in presence of KS. Holo-ACP is shown as reference. No depletion of HB-ACP nor malonyl-ACP can be seen.

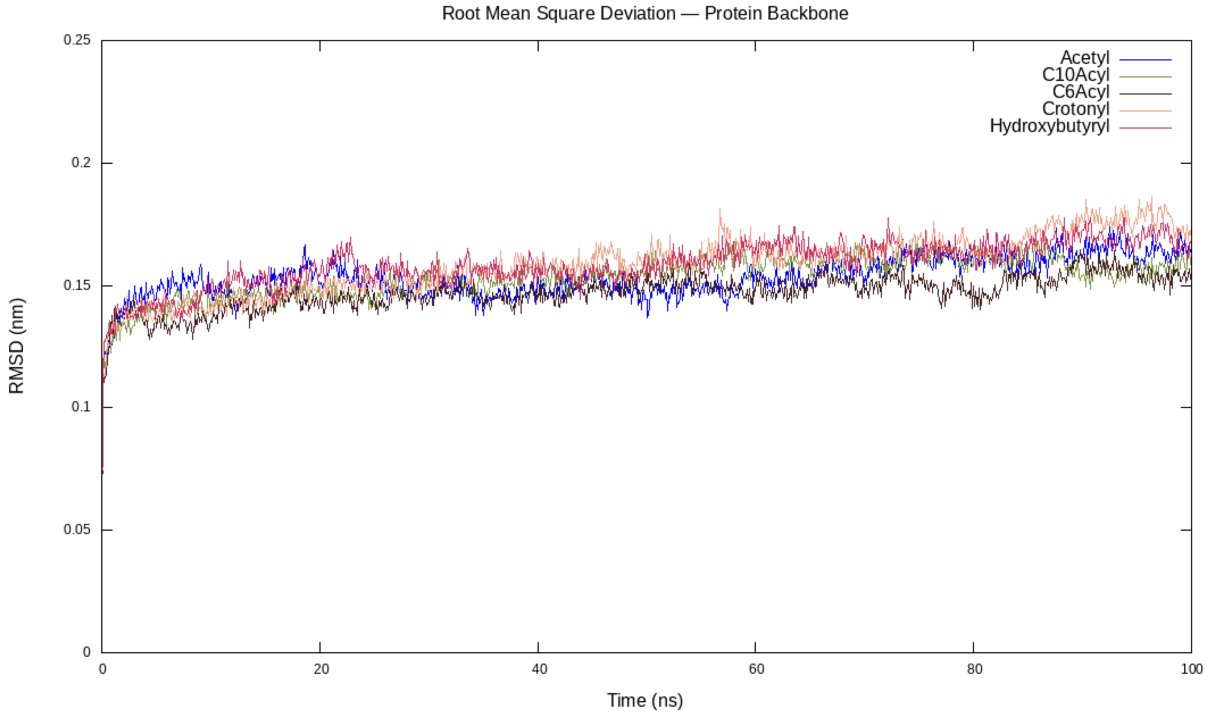

Figure S21: The Root Mean Square Deviation (RMSD) of the KS protein backbone in each KS:substrate complex throughout the 100 ns simulations. The KS:acetyl-Ppant complex is colored in blue, KS:C10acyl-Ppant complex in green, KS:C6acyl-Ppant complex in brown, KS:crotonyl-Ppant complex in pink and KS:hydroxybutyryl-Ppant in magenta. RMSD values are represented in nm. The positional restraints that assured the substrate initial position were released in the 50 ns mark.

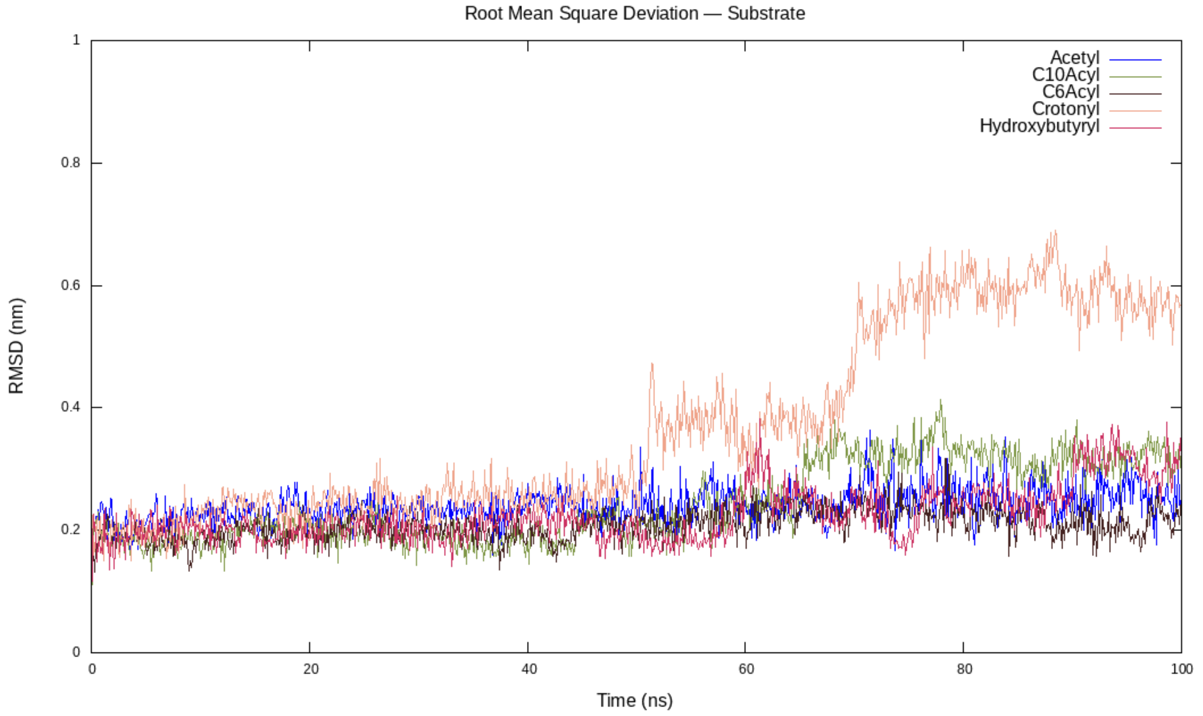

Figure S22: Root Mean Square Deviation (RMSD) of each substrate in every KS:substrate complex throughout the 100 ns simulation. The KS:acetyl-Ppant complex is colored in blue, the KS:C10acyl-Ppant complex in green, the KS:C6acyl-Ppant complex in brown, the KS:crotonyl-Ppant complex in pink, and the KS:hydroxybutyryl-Ppant in magenta. RMSD values are represented in nanometers. It is worth noting that the positional restraints that assured the substrate initial position were released in the 50 ns mark.

Upon releasing the atomic constraints at 50 ns, the non-substrate crotonyl shifts its position within the binding site. This reorientation in the dihedral angle of the bond between the PNS and crotonyl weakens the interactions with the catalytic residues and elongates the hydrogen bonds in the oxyanion hole. These changes cause the

carbonyl oxygen of the crotonyl group to lose its proper orientation toward the oxyanion hole in the enzyme's active site. As a result, the nucleophilic cysteine (Cys161), which is responsible for the nucleophilic attack, also becomes more distanced from the carbonyl carbon of crotonyl. The conformational change of the carbonyl group and the increased distance of the cysteine hinder the nucleophilic attack on the carbon of the bond to be cleaved; over some nanoseconds with these interactions disrupted, the crotonyl substrate significantly shifts its position in the active site. This results in the substrate moving away from the optimal position for catalysis. With the change in crotonyl's position, Phe395 also undergoes a structural rearrangement and adjusts its position to interact with the double bond of crotonyl. The rearrangement stems from structural differences between crotonyl and the natural substrates, providing a rationale for its diminished reactivity.

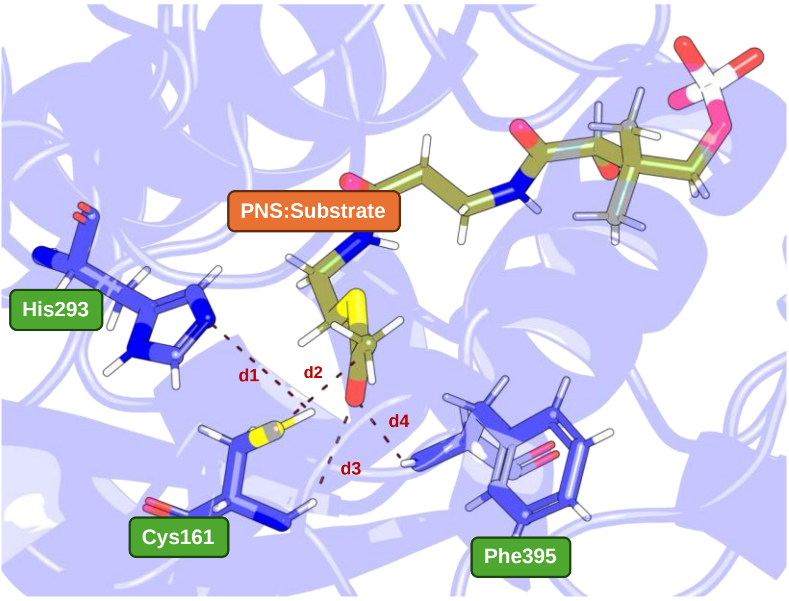

Figure S23: Visual representation of the four analyzed distances: two catalytic distances and two hydrogen bonds within the oxyanion hole. The Ppant:Substrate is colored green, and the residues that comprise the reactive site of the KS domain are colored purple. Additionally, each distance is represented by red dashed lines. The figure provides a structural overview highlighting key molecular interactions critical for enzyme function. Distance d1 denotes the proximity between Cys161(SH) and His293(Nε), necessary for thiol deprotonation. Distance d2 illustrates the interaction between Cys161(S) and Ppant(C), indicative of the easiness of the subsequent nucleophilic attack. Distances d3 and d4 correspond to the hydrogen bond lengths of the oxyanion hole, which is crucial for stabilizing the transition state in which the carbonyl oxygen bears a marked negative charge.

List of Sequences:

Mutations are marked in yellow. Affinity tags (His/ Strep) with respective linker are marked in cyan.

Nucleic acid sequence of template plasmid KS-MAT^{S581A}:

The gene encoding for KS-MAT is marked with lowercasified letters, whereas the backbone is shown in capitalized letters.

TAATACGACTCACTATAGGGGAATTGTGAGCGGATAACAATTCCCCTCTAGAAATAATTTTGTTTA
ACTTTAAGAAGGAGATATACATATGAGCGCTTGGAGCCATCCACAATTTGAGAAGGGTGGAGGTT
CTGGCGGTGGATCGGGAGGTTCAGCGTGGAGCCACCCGCAGTTCGAAAAAGGCGCCGGAtccga
ggaggtggtgatagccggtatgtcggggaagttgcccgagtcagagaacctacaggagttctgggccaacctcattggtggtgtggacatggt
cacagatgatgacaggagatggaaggctgggctctatggattacccaagcggtctggaaagctgaaggatctctccaagttcgacgcctccttt
tttggggtccaccccaagcaggcacacacaatggacccccagcttcggctgctgttggaagtcagctatgaagcaattgtggatggaggtatc
aacccagcctcactccgaggaacgaacactggcgtctgggtgggtgtgagtggttcagaggcatccgaggcccttagcagagatcccgaga
cgcttctgggctacagcatggtgggctgccagcgtgcaatgatggccaaccggctctctttcttcttcgacttcaaaggaccaagcattgccctgg
acacagcctgctcctccagcttgctggcactacagaatgcctaccaggccatccgtagtggggaatgccccgcggcccttgtgggtgggatca
acctgctcctgaagccgaacacctctgtgcagttcatgaagctgggcatgctcagcccggacggcacctgcagatcctttgatgattcagggag
tggatattgtcgctctgaggctgttgtagcagttctgctgactaagaagtccctggctcggcgggtctatgccacgattctgaatgccggcaccaat
acagatggcagcaaggagcaaggtgtaacattcccctctggagaagtccaagaacaactcatctgctctctgtatcagccagctggtctggcc
ccggagtcgcttgagtatattgaagcccatggcacgggcaccaaggtgggtgacccccaggaactgaatggcattactcggtccctgtgcgcc
ttccgccaggcccctctgttaattggctccaccaaatccaacatgggacaccctgagcctgcctctgggcttgcagccctgaccaaggtgctgtt
atccctggagcatggggtctgggcccctaacctgcacttccacaaccccaaccctgagatcccagcacttcttgatgggcggctgcaggtggtc
gataggcccctgcctgttcgtggtggcaacgtgggcatcaactcatttggcttcggaggctccaatgttcatgtcatcctccagcccaacacacgg
caggcccctgcgcccactgcacacgctgcccttccccatttgctgcacgccagtggacgcaccttagaggcagtgcaggacctgctggaaca
gggccgccagcacagccaggacctggcctttgtgagcatgctcaatgacattgcggcaacccctacagcagccatgcccttcaggggttaca
ctgtgctaggtgttgagggccgtgtccaagaagtgcagcaagtgtccaccaacaagcgcccactctggttcatctgctcagggatgggcacgc
agtggcgcgggatggggctgagcctcatgcgcctggacagcttccgtgagtctatcctgcgctccgatgaggctgtgaagccgttgggagtga
aagtgtcagatctgctgttgagcacagatgagcgcacctttgatgacatcgtgcatgcctttgtgagcctcactgccatccagattgccctcatcga
cctactgacttctgtgggactgaaacctgacggcatcattgggcacGccttgggagaggttgcctgtggctatgcagatggctgtctctcccaga
gagaggctgtgcttgcagcttactggcgaggccagtgcatcaaagatgcccacctcccgcctggatccatggcagctgttggtttgtcctgggag
gaatgtaaacagcgctgccccgctggcgtggtgcctgcctgccacaactctgaggacaccgtgaccatctctggacctcaggctgcagtgaat
gaatttgtggagcagctaaagcaagaaggtgtgtttgccaaggaggtacgaacaggaggcctggctttccactcctacttcatggaaggaattg
cccccacattgctgcaggctctcaagaaggtgatccgggaaccacggccgcgctcggctcgatggctcagcacctctatccctgaggcccagt
ggcagagcagcctggcccgcacatcttctgccgagtacaatgtcaacaacctggtgagccctgtgctcttccaggaagcactgtggcacatcc
ctgagcatgccgtggtgctggagattgcgccccacgcactgttgcaggctgtcctgaagcgaggcgtgaagtccagctgcaccatcattccctt
gatgaagagggatcataaagataacttggagttctttctcaccaaccttggcaaggtgcacctcacaggcatcaatgtcaaccctaacgccttgt
tcccacctgtggagttcccggctccccgagggactcctctcatctcccctcacatcaagtgggaccacagtcagacttgggatgtcccggttgctg
aggacttcccaaacGGCTCAGGTTCCCCCTCAGCCCATCATCACCACCACCACCACTGAGATCCG
GCTGCTAACAAAGCCCGAAAGGAAGCTGAGTTGGCTGCTGCCACCGCTGAGCAATAACTAGCAT
AACCCCTTGGGGCCTCTAAACGGGTCTTGAGGGGTTTTTTGCTGAAAGGAGGAACTATATCCGG
ATTGGCGAATGGGACGCGCCCTGTAGCGGCGCATTAAGCGCGGCGGGTGTGGTGGTTACGCGC
AGCGTGACCGCTACACTTGCCAGCGCCCTAGCGCCCGCTCCTTTCGCTTTCTTCCCTTCCTTTCT
CGCCACGTTCGCCGGCTTTCCCCGTCAAGCTCTAAATCGGGGGCTCCCTTTAGGGTTCCGATTT
AGTGCTTTACGGCACCTCGACCCCAAAAAACTTGATTAGGGTGATGGTTCACGTAGTGGGCCATC
GCCCTGATAGACGGTTTTTCGCCCTTTGACGTTGGAGTCCACGTTCTTTAATAGTGGACTCTTGTT
CCAAACTGGAACAACACTCAACCCTATCTCGGTCTATTCTTTTGATTTATAAGGGATTTTGCCGAT
TTCGGCCTATTGGTTAAAAAATGAGCTGATTTAACAAAAATTTAACGCGAATTTTAACAAAATATTA
ACGTTTACAATTTCAGGTGGCACTTTTCGGGGAAATGTGCGCGGAACCCCTATTTGTTTATTTTTC
TAAATACATTCAAATATGTATCCGCTCATGAGACAATAACCCTGATAAATGCTTCAATAATATTGAA
AAAGGAAGAGTATGAGTATTCAACATTTCCGTGTCGCCCTTATTCCCTTTTTTGCGGCATTTTGCC
TTCCTGTTTTTGCTCACCCAGAAACGCTGGTGAAAGTAAAAGATGCTGAAGATCAGTTGGGTGCA
CGAGTGGGTTACATCGAACTGGATCTCAACAGCGGTAAGATCCTTGAGAGTTTTCGCCCCGAAG
AACGTTTTCCAATGATGAGCACTTTTAAAGTTCTGCTATGTGGCGCGGTATTATCCCGTATTGACG
CCGGGCAAGAGCAACTCGGTCGCCGCATACACTATTCTCAGAATGACTTGGTTGAGTACTCACCA
GTCACAGAAAAGCATCTTACGGATGGCATGACAGTAAGAGAATTATGCAGTGCTGCCATAACCAT
GAGTGATAACACTGCGGCCAACTTACTTCTGACAACGATCGGAGGACCGAAGGAGCTAACCGCT
TTTTTGCACAACATGGGGGATCATGTAACTCGCCTTGATCGTTGGGAACCGGAGCTGAATGAAGC
CATACCAAACGACGAGCGTGACACCACGATGCCTGCAGCAATGGCAACAACGTTGCGCAAACTA
TTAACTGGCGAACTACTTACTCTAGCTTCCCGGCAACAATTAATAGACTGGATGGAGGCGGATAA
AGTTGCAGGACCACTTCTGCGCTCGGCCCTTCCGGCTGGCTGGTTTATTGCTGATAAATCTGGA
GCCGGTGAGCGTGGGTCTCGCGGTATCATTGCAGCACTGGGGCCAGATGGTAAGCCCTCCCGT
ATCGTAGTTATCTACACGACGGGGAGTCAGGCAACTATGGATGAACGAAATAGACAGATCGCTGA

GATAGGTGCCTCACTGATTAAGCATTGGTAACTGTCAGACCAAGTTTACTCATATATACTTTAGAT
TGATTTAAAACTTCATTTTTAATTTAAAAGGATCTAGGTGAAGATCCTTTTTGATAATCTCATGACC
AAAATCCCTTAACGTGAGTTTTCGTTCCACTGAGCGTCAGACCCCGTAGAAAAGATCAAAGGATC
TTCTTGAGATCCTTTTTTTCTGCGCGTAATCTGCTGCTTGCAAACAAAAAAACCACCGCTACCAGC
GGTGGTTTGTTTGCCGGATCAAGAGCTACCAACTCTTTTTCCGAAGGTAACTGGCTTCAGCAGAG
CGCAGATACCAAATACTGTCCTTCTAGTGTAGCCGTAGTTAGGCCACCACTTCAAGAACTCTGTA
GCACCGCCTACATACCTCGCTCTGCTAATCCTGTTACCAGTGGCTGCTGCCAGTGGCGATAAGT
CGTGTCTTACCGGGTTGGACTCAAGACGATAGTTACCGGATAAGGCGCAGCGGTCGGGCTGAAC
GGGGGGTTCGTGCACACAGCCCAGCTTGGAGCGAACGACCTACACCGAACTGAGATACCTACA
GCGTGAGCTATGAGAAAGCGCCACGCTTCCCGAAGGGAGAAAGGCGGACAGGTATCCGGTAAG
CGGCAGGGTCGGAACAGGAGAGCGCACGAGGGAGCTTCCAGGGGGAAACGCCTGGTATCTTTA
TAGTCCTGTCGGGTTTCGCCACCTCTGACTTGAGCGTCGATTTTTGTGATGCTCGTCAGGGGGG
CGGAGCCTATGGAAAAACGCCAGCAACGCGGCCTTTTTACGGTTCCTGGCCTTTTGCTGGCCTTT
TGCTCACATGTTCTTTCCTGCGTTATCCCCTGATTCTGTGGATAACCGTATTACCGCCTTTGAGTG
AGCTGATACCGCTCGCCGCAGCCGAACGACCGAGCGCAGCGAGTCAGTGAGCGAGGAAGCGG
AAGAGCGCCTGATGCGGTATTTTCTCCTTACGCATCTGTGCGGTATTTCACACCGCATATATGGT
GCACTCTCAGTACAATCTGCTCTGATGCCGCATAGTTAAGCCAGTATACACTCCGCTATCGCTAC
GTGACTGGGTCATGGCTGCGCCCCGACACCCGCCAACACCCGCTGACGCGCCCTGACGGGCTT
GTCTGCTCCCGGCATCCGCTTACAGACAAGCTGTGACCGTCTCCGGGAGCTGCATGTGTCAGAG
GTTTTCACCGTCATCACCGAAACGCGCGAGGCAGCTGCGGTAAAGCTCATCAGCGTGGTCGTGA
AGCGATTCACAGATGTCTGCCTGTTCATCCGCGTCCAGCTCGTTGAGTTTCTCCAGAAGCGTTAA
TGTCTGGCTTCTGATAAAGCGGGCCATGTTAAGGGCGGTTTTTTCCTGTTTGGTCACTGATGCCT
CCGTGTAAGGGGGATTTCTGTTCATGGGGGTAATGATACCGATGAAACGAGAGAGGATGCTCAC
GATACGGGTTACTGATGATGAACATGCCCGGTTACTGGAACGTTGTGAGGGTAAACAACTGGCG
GTATGGATGCGGCGGGACCAGAGAAAAATCACTCAGGGTCAATGCCAGCGCTTCGTTAATACAG
ATGTAGGTGTTCCACAGGGTAGCCAGCAGCATCCTGCGATGCAGATCCGGAACATAATGGTGCA
GGGCGCTGACTTCCGCGTTTCCAGACTTTACGAAACACGGAAACCGAAGACCATTCATGTTGTTG
CTCAGGTCGCAGACGTTTTGCAGCAGCAGTCGCTTCACGTTCGCTCGCGTATCGGTGATTCATTC
TGCTAACCAGTAAGGCAACCCCGCCAGCCTAGCCGGGTCCTCAACGACAGGAGCACGATCATGC
GCACCCGTGGGGCCGCCATGCCGGCGATAATGGCCTGCTTCTCGCCGAAACGTTTGGTGGCGG
GACCAGTGACGAAGGCTTGAGCGAGGGCGTGCAAGATTCCGAATACCGCAAGCGACAGGCCGA
TCATCGTCGCGCTCCAGCGAAAGCGGTCCTCGCCGAAAATGACCCAGAGCGCTGCCGGCACCT
GTCCTACGAGTTGCATGATAAAGAAGACAGTCATAAGTGCGGCGACGATAGTCATGCCCCGCGC
CCACCGGAAGGAGCTGACTGGGTTGAAGGCTCTCAAGGGCATCGGTCGAGATCCCGGTGCCTA
ATGAGTGAGCTAACTTACATTAATTGCGTTGCGCTCACTGCCCGCTTTCCAGTCGGGAAACCTGT
CGTGCCAGCTGCATTAATGAATCGGCCAACGCGCGGGGAGAGGCGGTTTGCGTATTGGGCGCC
AGGGTGGTTTTTCTTTTCACCAGTGAGACGGGCAACAGCTGATTGCCCTTCACCGCCTGGCCCT
GAGAGAGTTGCAGCAAGCGGTCCACGCTGGTTTGCCCCAGCAGGCGAAAATCCTGTTTGATGGT
GGTTAACGGCGGGATATAACATGAGCTGTCTTCGGTATCGTCGTATCCCACTACCGAGATATCCG
CACCAACGCGCAGCCCGGACTCGGTAATGGCGCGCATTGCGCCCAGCGCCATCTGATCGTTGG
CAACCAGCATCGCAGTGGGAACGATGCCCTCATTCAGCATTTGCATGGTTTGTTGAAAACCGGAC
ATGGCACTCCAGTCGCCTTCCCGTTCCGCTATCGGCTGAATTTGATTGCGAGTGAGATATTTATG
CCAGCCAGCCAGACGCAGACGCGCCGAGACAGAACTTAATGGGCCCGCTAACAGCGCGATTTG
CTGGTGACCCAATGCGACCAGATGCTCCACGCCCAGTCGCGTACCGTCTTCATGGGAGAAAATA
ATACTGTTGATGGGTGTCTGGTCAGAGACATCAAGAAATAACGCCGGAACATTAGTGCAGGCAG
CTTCCACAGCAATGGCATCCTGGTCATCCAGCGGATAGTTAATGATCAGCCCACTGACGCGTTGC
GCGAGAAGATTGTGCACCGCCGCTTTACAGGCTTCGACGCCGCTTCGTTCTACCATCGACACCA
CCACGCTGGCACCCAGTTGATCGGCGCGAGATTTAATCGCCGCGACAATTTGCGACGGCGCGT
GCAGGGCCAGACTGGAGGTGGCAACGCCAATCAGCAACGACTGTTTGCCCGCCAGTTGTTGTG
CCACGCGGTTGGGAATGTAATTCAGCTCCGCCATCGCCGCTTCCACTTTTTCCCGCGTTTTCGCA
GAAACGTGGCTGGCCTGGTTCACCACGCGGGAAACGGTCTGATAAGAGACACCGGCATACTCTG
CGACATCGTATAACGTTACTGGTTTCACATTCACCACCCTGAATTGACTCTCTTCCGGGCGCTATC
ATGCCATACCGCGAAAGGTTTTGCGCCATTCGATGGTGTCCGGGATCTCGACGCTCTCCCTTATG
CGACTCCTGCATTAGGAAGCAGCCCAGTAGTAGGTTGAGGCCGTTGAGCACCGCCGCCGCAAG
GAATGGTGCATGCAAGGAGATGGCGCCCAACAGTCCCCCGGCCACGGGGCCTGCCACCATACC
CACGCCGAAACAAGCGCTCATGAGCCCGAAGTGGCGAGCCCGATCTTCCCCATCGGTGATGTC
GGCGATATAGGCGCCAGCAACCGCACCTGTGGCGCCGGTGATGCCGGCCACGATGCGTCCGG
CGTAGAGGATCGAGATCTCGATCCCGCGAAAT

Amino acid sequence of MabA
MHHHHHHHHTATATEGAKPPFVSRSVLVTGGNRGIGLAIAQRLAADGHKVAVTHRGSGAP
KGLFGVECDVTDSDAVDRAFTAVEEHQGPVEVLVSNAGLSADAFLMRMTEEKFEKVINANL
TGAFRVAQRASRSMQRNKFGRMIFIGSVSGSWGIGNQANYAASKAGVIGMARSIARELSKA
NVTANVVAPGYIDTDMTRALDERIQQGALQFIPAKRVGTPAEVAGVVSFLASEDASYISGAVI
PVDGGMGMGH
The plasmid is based on the pET22 vector and was prepared by Alexander Rittner.

Amino acid sequence of ACP
MSAWSHPQFEKGAGDGDTQRDLVKAVAHILGIRDLAGINLDSTLADLGLDSLMGVEVRQILE
REHDLVLPMREVRQLTLRKLQEMSSKTDSATDTTAPLEHHHHHHHH
The plasmid is based on the pET22 vector and was prepared by Alexander Rittner.[42]

Amino acid sequence of Sfp
MKIYGIYMDRPLSQEENERFMTFISPEKREKCRRFYHKEDAHRTLLGDVLVRSVISRQYQLD
KSDIRFSTQEYGKPCIPDLPDAHFNISHSGRWVIGAFDSQPIGIDIEKTKPISLEIAKRFFSKTE
YSDLLAKDKDEQTDYFYHLWSMKESFIKQEGKGLSLPLDSFSVRLHQDGQVSIELPDSHSP
CYIKTYEVDPGYKMAVCAAHPDFPEDITMVSYEELLRSHHHHHH
The plasmid is based on the pQE60 vector and was prepared by Peter Tufar.[76]

Amino acid sequence of KS-MAT^{S581A}
MSAWSHPQFEKGGGSGGGSGGSAWSHPQFEKGAGSEEVVIAGMSGKLPESENLQEFWA
NLIGGVDMVTDDDRRWKAGLYGLPKRSGKLKDLSKFDASFFGVHPKQAHTMDPQLRLLLE
VSYEAIVDGGINPASLRGTNTGVWVGVSGSEASEALSRDPETLLGYSMVGCQRAMMANRL
SFFFDFKGPSIALDTACSSSLLALQNAYQAIRSGECPAALVGGINLLLKPNTSVQFMKLGMLS
PDGTCRSFDDSGSGYCRSEAVVAVLLTKKSLARRVYATILNAGTNTDGSKEQGVTFPSGEV
QEQLICSLYQPAGLAPESLEYIEAHGTGTKVGDPQELNGITRSLCAFRQAPLLIGSTKSNMG
HPEPASGLAALTKVLLSLEHGVWAPNLHFHNPNPEIPALLDGRLQVVDRPLPVRGGNVGINS
FGFGGSNVHVILQPNTRQAPAPTAHAALPHLLHASGRTLEAVQDLLEQGRQHSQDLAFVSM
LNDIAATPTAAMPFRGYTVLGVEGRVQEVQQVSTNKRPLWFICSGMGTQWRGMGLSLMRL
DSFRESILRSDEAVKPLGVKVSDLLLSTDERTFDDIVHAFVSLTAIQIALIDLLTSVGLKPDGIIG
HALGEVACGYADGCLSQREAVLAAYWRGQCIKDAHLPPGSMAAVGLSWEECKQRCPAGV
VPACHNSEDTVTISGPQAAVNEFVEQLKQEGVFAKEVRTGGLAFHSYFMEGIAPTLLQALKK
VIREPRPRSARWLSTSIPEAQWQSSLARTSSAEYNVNNLVSPVLFQEALWHIPEHAVVLEIA
PHALLQAVLKRGVKSSCTIIPLMKRDHKDNLEFFLTNLGKVHLTGINVNPNALFPPVEFPAPR
GTPLISPHIKWDHSQTWDVPVAEDFPNGSGSPSAHHHHHHHH
The plasmid is based on the pET22 vector and was prepared by Alexander Rittner.[24]

Amino acid sequence of KS^{C161G}-MAT^{S581A}
MSAWSHPQFEKGGGSGGGSGGSAWSHPQFEKGAGSEEVVIAGMSGKLPESENLQEFWA
NLIGGVDMVTDDDRRWKAGLYGLPKRSGKLKDLSKFDASFFGVHPKQAHTMDPQLRLLLE
VSYEAIVDGGINPASLRGTNTGVWVGVSGSEASEALSRDPETLLGYSMVGCQRAMMANRL
SFFFDFKGPSIALDTAGSSSLLALQNAYQAIRSGECPAALVGGINLLLKPNTSVQFMKLGMLS
PDGTCRSFDDSGSGYCRSEAVVAVLLTKKSLARRVYATILNAGTNTDGSKEQGVTFPSGEV
QEQLICSLYQPAGLAPESLEYIEAHGTGTKVGDPQELNGITRSLCAFRQAPLLIGSTKSNMG
HPEPASGLAALTKVLLSLEHGVWAPNLHFHNPNPEIPALLDGRLQVVDRPLPVRGGNVGINS
FGFGGSNVHVILQPNTRQAPAPTAHAALPHLLHASGRTLEAVQDLLEQGRQHSQDLAFVSM
LNDIAATPTAAMPFRGYTVLGVEGRVQEVQQVSTNKRPLWFICSGMGTQWRGMGLSLMRL
DSFRESILRSDEAVKPLGVKVSDLLLSTDERTFDDIVHAFVSLTAIQIALIDLLTSVGLKPDGIIG
HALGEVACGYADGCLSQREAVLAAYWRGQCIKDAHLPPGSMAAVGLSWEECKQRCPAGV
VPACHNSEDTVTISGPQAAVNEFVEQLKQEGVFAKEVRTGGLAFHSYFMEGIAPTLLQALKK
VIREPRPRSARWLSTSIPEAQWQSSLARTSSAEYNVNNLVSPVLFQEALWHIPEHAVVLEIA
PHALLQAVLKRGVKSSCTIIPLMKRDHKDNLEFFLTNLGKVHLTGINVNPNALFPPVEFPAPR
GTPLISPHIKWDHSQTWDVPVAEDFPNGSGSPSAHHHHHHHH

Amino acid sequence of KS^{D158N}-MAT^{S581A}

MSAWSHPQFEKGGGSGGGSGGSAWSHPQFEKGAGSEEVVIAGMSGKLPESENLQEFWA
NLIGGVDMVTDDDRRWKAGLYGLPKRSGKLKDLSKFDASFFGVHPKQAHTMDPQLRLLLE
VSYEAIVDGGINPASLRGTNTGVWVGVSGSEASEALSRDPETLLGYSMVGCQRAMMANRL
SFFFDFKGPSIALNTACSSSLLALQNAYQAIRSGECPAALVGGINLLLKPNTSVQFMKLGMLS
PDGTCRSFDDSGSGYCRSEAVVAVLLTKKSLARRVYATILNAGTNTDGSKEQGVTFPSGEV
QEQLICSLYQPAGLAPESLEYIEAHGTGTKVGDPQELNGITRSLCAFRQAPLLIGSTKSNMG
HPEPASGLAALTKVLLSLEHGVWAPNLHFHNPNPEIPALLDGRLQVVDRPLPVRGGNVGINS
FGFGGSNVHVILQPNTRQAPAPTAHAALPHLLHASGRTLEAVQDLLEQGRQHSQDLAFVSM
LNDIAATPTAAMPFRGYTVLGVEGRVQEVQQVSTNKRPLWFICSGMGTQWRGMGLSLMRL
DSFRESILRSDEAVKPLGVKVSDLLLSTDERTFDDIVHAFVSLTAIQIALIDLLTSVGLKPDGIIG
HALGEVACGYADGCLSQREAVLAAYWRGQCIKDAHLPPGSMAAVGLSWEECKQRCPAGV
VPACHNSEDTVTISGPQAAVNEFVEQLKQEGVFAKEVRTGGLAFHSYFMEGIAPTLLQALKK
VIREPRPRSARWLSTSIPEAQWQSSLARTSSAEYNVNNLVSPVLFQEALWHIPEHAVVLEIA
PHALLQAVLKRGVKSSCTIIPLMKRDHKDNLEFFLTNLGKVHLTGINVNPNALFPPVEFPAPR
GTPLISPHIKWDHSQTWDVPVAEDFPNGSGSPSAHHHHHHHH

Amino acid sequence of KS^{D158S}-MAT^{S581A}

MSAWSHPQFEKGGGSGGGSGGSAWSHPQFEKGAGSEEVVIAGMSGKLPESENLQEFWA
NLIGGVDMVTDDDRRWKAGLYGLPKRSGKLKDLSKFDASFFGVHPKQAHTMDPQLRLLLE
VSYEAIVDGGINPASLRGTNTGVWVGVSGSEASEALSRDPETLLGYSMVGCQRAMMANRL
SFFFDFKGPSIALSTACSSSLLALQNAYQAIRSGECPAALVGGINLLLKPNTSVQFMKLGMLS
PDGTCRSFDDSGSGYCRSEAVVAVLLTKKSLARRVYATILNAGTNTDGSKEQGVTFPSGEV
QEQLICSLYQPAGLAPESLEYIEAHGTGTKVGDPQELNGITRSLCAFRQAPLLIGSTKSNMG
HPEPASGLAALTKVLLSLEHGVWAPNLHFHNPNPEIPALLDGRLQVVDRPLPVRGGNVGINS
FGFGGSNVHVILQPNTRQAPAPTAHAALPHLLHASGRTLEAVQDLLEQGRQHSQDLAFVSM
LNDIAATPTAAMPFRGYTVLGVEGRVQEVQQVSTNKRPLWFICSGMGTQWRGMGLSLMRL
DSFRESILRSDEAVKPLGVKVSDLLLSTDERTFDDIVHAFVSLTAIQIALIDLLTSVGLKPDGIIG
HALGEVACGYADGCLSQREAVLAAYWRGQCIKDAHLPPGSMAAVGLSWEECKQRCPAGV
VPACHNSEDTVTISGPQAAVNEFVEQLKQEGVFAKEVRTGGLAFHSYFMEGIAPTLLQALKK
VIREPRPRSARWLSTSIPEAQWQSSLARTSSAEYNVNNLVSPVLFQEALWHIPEHAVVLEIA
PHALLQAVLKRGVKSSCTIIPLMKRDHKDNLEFFLTNLGKVHLTGINVNPNALFPPVEFPAPR
GTPLISPHIKWDHSQTWDVPVAEDFPNGSGSPSAHHHHHHHH

Amino acid sequence of KS^{R137K}-MAT^{S581A}

MSAWSHPQFEKGGGSGGGSGGSAWSHPQFEKGAGSEEVVIAGMSGKLPESENLQEFWA
NLIGGVDMVTDDDRRWKAGLYGLPKRSGKLKDLSKFDASFFGVHPKQAHTMDPQLRLLLE
VSYEAIVDGGINPASLRGTNTGVWVGVSGSEASEALSRDPETLLGYSMVGCQKAMMANRL
SFFFDFKGPSIALDTACSSSLLALQNAYQAIRSGECPAALVGGINLLLKPNTSVQFMKLGMLS
PDGTCRSFDDSGSGYCRSEAVVAVLLTKKSLARRVYATILNAGTNTDGSKEQGVTFPSGEV
QEQLICSLYQPAGLAPESLEYIEAHGTGTKVGDPQELNGITRSLCAFRQAPLLIGSTKSNMG
HPEPASGLAALTKVLLSLEHGVWAPNLHFHNPNPEIPALLDGRLQVVDRPLPVRGGNVGINS
FGFGGSNVHVILQPNTRQAPAPTAHAALPHLLHASGRTLEAVQDLLEQGRQHSQDLAFVSM
LNDIAATPTAAMPFRGYTVLGVEGRVQEVQQVSTNKRPLWFICSGMGTQWRGMGLSLMRL
DSFRESILRSDEAVKPLGVKVSDLLLSTDERTFDDIVHAFVSLTAIQIALIDLLTSVGLKPDGIIG
HALGEVACGYADGCLSQREAVLAAYWRGQCIKDAHLPPGSMAAVGLSWEECKQRCPAGV
VPACHNSEDTVTISGPQAAVNEFVEQLKQEGVFAKEVRTGGLAFHSYFMEGIAPTLLQALKK
VIREPRPRSARWLSTSIPEAQWQSSLARTSSAEYNVNNLVSPVLFQEALWHIPEHAVVLEIA
PHALLQAVLKRGVKSSCTIIPLMKRDHKDNLEFFLTNLGKVHLTGINVNPNALFPPVEFPAPR
GTPLISPHIKWDHSQTWDVPVAEDFPNGSGSPSAHHHHHHHH

Amino acid sequence of KS^{R137A}-MAT^{S581A}

MSAWSHPQFEKGGGSGGGSGGSAWSHPQFEKGAGSEEVVIAGMSGKLPESENLQEFWA
NLIGGVDMVTDDDRRWKAGLYGLPKRSGKLKDLSKFDASFFGVHPKQAHTMDPQLRLLLE
VSYEAIVDGGINPASLRGTNTGVWVGVSGSEASEALSRDPETLLGYSMVGCQAAMMANRL
SFFFDFKGPSIALDTACSSSLLALQNAYQAIRSGECPAALVGGINLLLKPNTSVQFMKLGMLS
PDGTCRSFDDSGSGYCRSEAVVAVLLTKKSLARRVYATILNAGTNTDGSKEQGVTFPSGEV
QEQLICSLYQPAGLAPESLEYIEAHGTGTKVGDPQELNGITRSLCAFRQAPLLIGSTKSNMG
HPEPASGLAALTKVLLSLEHGVWAPNLHFHNPNPEIPALLDGRLQVVDRPLPVRGGNVGINS
FGFGGSNVHVILQPNTRQAPAPTAHAALPHLLHASGRTLEAVQDLLEQGRQHSQDLAFVSM
LNDIAATPTAAMPFRGYTVLGVEGRVQEVQQVSTNKRPLWFICSGMGTQWRGMGLSLMRL
DSFRESILRSDEAVKPLGVKVSDLLLSTDERTFDDIVHAFVSLTAIQIALIDLLTSVGLKPDGIIG
HALGEVACGYADGCLSQREAVLAAYWRGQCIKDAHLPPGSMAAVGLSWEECKQRCPAGV
VPACHNSEDTVTISGPQAAVNEFVEQLKQEGVFAKEVRTGGLAFHSYFMEGIAPTLLQALKK
VIREPRPRSARWLSTSIPEAQWQSSLARTSSAEYNVNNLVSPVLFQEALWHIPEHAVVLEIA
PHALLQAVLKRGVKSSCTIIPLMKRDHKDNLEFFLTNLGKVHLTGINVNPNALFPPVEFPAPR
GTPLISPHIKWDHSQTWDVPVAEDFPNGSGSPSAHHHHHHHH

Amino acid sequence of KS^{A160G}-MAT^{S581A}

MSAWSHPQFEKGGGSGGGSGGSAWSHPQFEKGAGSEEVVIAGMSGKLPESENLQEFWA
NLIGGVDMVTDDDRRWKAGLYGLPKRSGKLKDLSKFDASFFGVHPKQAHTMDPQLRLLLE
VSYEAIVDGGINPASLRGTNTGVWVGVSGSEASEALSRDPETLLGYSMVGCQRAMMANRL
SFFFDFKGPSIALDTGCSSSLLALQNAYQAIRSGECPAALVGGINLLLKPNTSVQFMKLGMLS
PDGTCRSFDDSGSGYCRSEAVVAVLLTKKSLARRVYATILNAGTNTDGSKEQGVTFPSGEV
QEQLICSLYQPAGLAPESLEYIEAHGTGTKVGDPQELNGITRSLCAFRQAPLLIGSTKSNMG
HPEPASGLAALTKVLLSLEHGVWAPNLHFHNPNPEIPALLDGRLQVVDRPLPVRGGNVGINS
FGFGGSNVHVILQPNTRQAPAPTAHAALPHLLHASGRTLEAVQDLLEQGRQHSQDLAFVSM
LNDIAATPTAAMPFRGYTVLGVEGRVQEVQQVSTNKRPLWFICSGMGTQWRGMGLSLMRL
DSFRESILRSDEAVKPLGVKVSDLLLSTDERTFDDIVHAFVSLTAIQIALIDLLTSVGLKPDGIIG
HALGEVACGYADGCLSQREAVLAAYWRGQCIKDAHLPPGSMAAVGLSWEECKQRCPAGV
VPACHNSEDTVTISGPQAAVNEFVEQLKQEGVFAKEVRTGGLAFHSYFMEGIAPTLLQALKK
VIREPRPRSARWLSTSIPEAQWQSSLARTSSAEYNVNNLVSPVLFQEALWHIPEHAVVLEIA
PHALLQAVLKRGVKSSCTIIPLMKRDHKDNLEFFLTNLGKVHLTGINVNPNALFPPVEFPAPR
GTPLISPHIKWDHSQTWDVPVAEDFPNGSGSPSAHHHHHHHH

Amino acid sequence of KS^{A160V}-MAT^{S581A}

MSAWSHPQFEKGGGSGGGSGGSAWSHPQFEKGAGSEEVVIAGMSGKLPESENLQEFWA
NLIGGVDMVTDDDRRWKAGLYGLPKRSGKLKDLSKFDASFFGVHPKQAHTMDPQLRLLLE
VSYEAIVDGGINPASLRGTNTGVWVGVSGSEASEALSRDPETLLGYSMVGCQRAMMANRL
SFFFDFKGPSIALDTVCSSSLLALQNAYQAIRSGECPAALVGGINLLLKPNTSVQFMKLGMLS
PDGTCRSFDDSGSGYCRSEAVVAVLLTKKSLARRVYATILNAGTNTDGSKEQGVTFPSGEV
QEQLICSLYQPAGLAPESLEYIEAHGTGTKVGDPQELNGITRSLCAFRQAPLLIGSTKSNMG
HPEPASGLAALTKVLLSLEHGVWAPNLHFHNPNPEIPALLDGRLQVVDRPLPVRGGNVGINS
FGFGGSNVHVILQPNTRQAPAPTAHAALPHLLHASGRTLEAVQDLLEQGRQHSQDLAFVSM
LNDIAATPTAAMPFRGYTVLGVEGRVQEVQQVSTNKRPLWFICSGMGTQWRGMGLSLMRL
DSFRESILRSDEAVKPLGVKVSDLLLSTDERTFDDIVHAFVSLTAIQIALIDLLTSVGLKPDGIIG
HALGEVACGYADGCLSQREAVLAAYWRGQCIKDAHLPPGSMAAVGLSWEECKQRCPAGV
VPACHNSEDTVTISGPQAAVNEFVEQLKQEGVFAKEVRTGGLAFHSYFMEGIAPTLLQALKK
VIREPRPRSARWLSTSIPEAQWQSSLARTSSAEYNVNNLVSPVLFQEALWHIPEHAVVLEIA
PHALLQAVLKRGVKSSCTIIPLMKRDHKDNLEFFLTNLGKVHLTGINVNPNALFPPVEFPAPR
GTPLISPHIKWDHSQTWDVPVAEDFPNGSGSPSAHHHHHHHH

List of primers

prCG048_KS_D158N_f	ccaagcattgccctgAacacagcctgctcctccag
prCG050_KS_D158S_f	ccaagcattgccctgTCcacagcctgctcctccag
prCG051_KS_R137K_f	atggtgggctgccagAAAgcaatgatggccaaccggc
prCG053_KS_R137A_f	atggtgggctgccagGCGgcaatgatggccaaccggc
prCG054_KS_A160G_f	cattgccctggacacagGctgctcctccagcttgctgg
prCG056_KS_A160V_f	cattgccctggacacagTctgctcctccagcttgctgg
prCG057_KS_D158S_r	ctggaggagcaggctgtgGAcagggcaatgcttgg
prCG058_KS_D158N_r	ctggaggagcaggctgtgtTcagggcaatgcttgg
prCG059_KS_R137K_r	gccggttggccatcattgcTTTctggcagcccaccat
prCG060_KS_R137A_r	gccggttggccatcattgcCGCctggcagcccaccat
prCG061_KS_A160G_r	ccagcaagctggaggagcagCctgtgtccagggcaatg
prCG062_KS_A160V_r	ccagcaagctggaggagcagActgtgtccagggcaatg
